# Etifoxine drives macrophage M2 polarization via Schwann cell-derived progesterone activation of PPARγ to accelerate peripheral nerve repair

**DOI:** 10.3389/fncel.2026.1789450

**Published:** 2026-03-23

**Authors:** Chao Guo, Song Liu

**Affiliations:** 1 Beijing Key Laboratory of Central Nervous System Injury, Beijing Neurosurgical Institute, Capital Medical University, Beijing, China; 2 U1195, Inserm et Universite Paris-Saclay, Le Kremlin-Bicetre, France

**Keywords:** M2 macrophage polarization, metabolism reprograming, peripheral nerve injury, Schwann cell, TSPO

## Abstract

**Background:**

Peripheral nerve injury (PNI) presents a significant clinical challenge due to limited endogenous regenerative capacity. The translocator protein (TSPO) ligand etifoxine (ETX) has shown promise in promoting nerve repair, but the underlying cellular and molecular mechanisms remain incompletely understood.

**Methods:**

Utilizing in vitro co-culture systems with human Schwann cells (HSCs) and THP-1-derived macrophages, TSPO-knockdown HSCs, conditioned medium experiments, and an in vivo rat sciatic nerve crush injury model, we investigated the effects of ETX on cellular crosstalk and macrophage polarization. Molecular analyses included RNA sequencing, western blotting, fatty acid oxidation (FAO) assays, and a Mito-QC reporter system to assess mitophagy. Functional recovery was evaluated through behavioral tests (hindlimb grip strength, mechanical pain threshold), immunofluorescence, and retrograde tracing.

**Results:**

ETX specifically activated TSPO on Schwann cells, stimulating progesterone synthesis and secretion. This Schwann cell-derived progesterone acted as a paracrine signal on macrophages, activating the PPARγ–PGC1α axis. This activation triggered dual reprogramming in macrophages: a metabolic shift toward FAO and induction of BNIP3L-mediated mitophagy, both essential for sustaining a pro-regenerative M2 phenotype. These effects were significantly attenuated by the progesterone receptor antagonist RU486 or the PPARγ antagonist GW9662. In vivo, ETX treatment accelerated functional recovery, enhanced axonal regeneration, and increased infiltration of M2 macrophages at the injury site, effects that were partially reversed by RU486 or GW9662 co-administration.

**Conclusion:**

ETX facilitates peripheral nerve repair by promoting Schwann cell-derived progesterone, which drives macrophage PPARγ pathway activation, orchestrating metabolic-autophagic reprogramming necessary for sustained M2 polarization. These findings identify a novel Schwann cell–macrophage metabolic crosstalk mechanism and support the therapeutic potential of targeting this axis in PNI.

## Highlights

- Etifoxine activates TSPO on Schwann cells, stimulating progesterone secretion.
- Schwann cell-derived progesterone activates macrophage PPARγ, driving metabolic reprograming toward fatty acid oxidation and inducing mitophagy.
- This metabolic-autophagic synergy sustains M2 macrophage polarization, accelerating functional and structural peripheral nerve repair.

## Introduction

Peripheral nerve injury (PNI), which results from traumatic accidents, iatrogenic injuries, and other etiologies (Brosius Lutz et al., 2022), represents a growing clinical burden worldwide. Epidemiological studies indicate an annual incidence of 13–23 cases per 100,000 persons in developed nations (Soto et al., 2021). Although peripheral nerves exhibit a regenerative capacity superior to that of their central counterparts (Mahar and Cavalli, 2018), their inherent repair mechanisms remain insufficient for functional restoration (Li J. et al., 2022). This regenerative limitation frequently manifests as partial or complete functional impairment in denervated regions, potentially progressing to permanent disability that profoundly compromises patients’ quality of life (Li et al., 2021).

Peripheral nerves harbor two distinct macrophage populations: resident macrophages, which maintain tissue homeostasis under steady-state conditions, and infiltrating macrophages, which are recruited during injury or infection responses (Zigmond and Echevarria, 2019). In adult rat sciatic nerves, resident macrophages constitute a minor fraction (2–9%) of the total cells (Gaudet et al., 2011). Infiltrating macrophages are derived from bone marrow monocytes that extravasate into tissues in response to chemotactic cytokines released by injured nerves (Mildner et al., 2016). A temporal analysis revealed that macrophage infiltration commences in distal nerve segments at 2–3 days post-injury, peaks on day 6 (Liu et al., 2019). M2 macrophages predominate during the later stages of peripheral nerve injury and persist long term (Nadeau et al., 2011), mediating tissue remodeling, repair, and healing processes (Viola et al., 2019). Macrophages promote peripheral nerve repair through three synergistic mechanisms: (1) the hypoxia response mediated by HIF-1α stabilization induces VEGF secretion to drive *angiogenesis*, restoring ischemic microenvironments; (2) Schwann cell support by M2a-derived IL-10 and TGF-β enhances *Schwann cell proliferation, migration, and remyelination*, with macrophage-guided vascular networks providing migratory scaffolds; and (3) inflammation–debris homeostasis is achieved through M1-mediated *phagocytic clearance* of necrotic debris and M2/M2c-secreted anti-inflammatory factors (e.g., IL-10) that *suppress chronic inflammation* while promoting *tissue remodeling*. This tripartite functional coordination creates a regenerative niche essential for axonal regeneration (Liu et al., 2019).

Translocator protein 18 kDa (TSPO) is an integral membrane protein primarily localized to the mitochondrial outer membrane (Lacapère and Papadopoulos, 2003), with its expression predominantly restricted to glial cell types, including ependymal cells, microglia, and astrocytes (Bonsack et al., 2016). TSPO participates in various cellular physiological processes, and substantial evidence indicates its critical role in neurosteroidogenesis. Specifically, TSPO binds cholesterol—the substrate for neurosteroid synthesis—and facilitates its transport across the mitochondrial membrane to the inner membrane, thereby initiating neurosteroid biosynthesis (Papadopoulos et al., 2006). Studies have shown that the TSPO ligand etifoxine (ETX) can enhance peripheral nerve regeneration and inhibit neuroinflammation by increasing the synthesis of pregnenolone and progesterone, and by suppressing macrophage activation (Girard et al., 2008). However, given the extremely limited capacity of macrophages to synthesize neurosteroids *de novo*, the precise mechanisms underlying ETX-induced neurosteroidogenesis—particularly the cellular communication pathways involved—remain unknown. In the present study, we demonstrate that ETX activates TSPO in Schwann cells, stimulating them to secrete progesterone. This Schwann cell-derived progesterone then acts as a primary paracrine signal on macrophages, leading to the activation of their PPARγ–PGC1α axis. We identify this axis as a central mechanism driving sustained M2 polarization, which in turn promotes metabolic reprograming in macrophages toward fatty acid oxidation and concurrently induces mitophagy. Given the multi-target pharmacology of ETX, we also explored potential ancillary contributions from GABA_A receptor signaling and progesterone metabolites, recognizing that nerve regeneration *in vivo* likely involves a complex regulatory network. This metabolic-autophagic synergy plays a crucial role in maintaining the M2 polarization state, ultimately supporting the structural and functional repair of peripheral nerves.

## Materials and methods

### Cell culture and coculture

In this study, human Schwann cells (HSCs) were primary cells purchased from Hefei Wanwu Biotechnology Co., Ltd. (Hefei, China). THP-1 cells are a human monocyte cell line obtained from ATCC. THP-1 monocytes were cultured in RPMI-1,640 medium supplemented with 10% fetal bovine serum (FBS) and 1% penicillin–streptomycin (P/S) at a density of 2–8 × 10^5^ cells/mL, and were passaged every 2–3 days. For M0 macrophage polarization, cells were seeded at 2–5 × 10^5^ cells/cm^2^, treated with 100 nM phorbol 12-myristate 13-acetate (PMA) for 48 h, washed with phosphate-buffered saline (PBS), and then maintained in PMA-free medium for an additional 24 h. HSCs were expanded in poly-L-lysine-coated culture vessels using Schwann cell medium (SCM), which consists of DMEM basal medium supplemented with 10% FBS, 1% Schwann cell growth supplement (ScienCell Resarch Laboretories), and 1% P/S. Upon reaching 90% confluence, cells were dissociated with trypsin/EDTA, washed, neutralized, and centrifuged at 1,000 × g for 5 min. For the coculture experiments, M0 macrophages were seeded in the lower chamber and HSCs in the upper chamber of a Transwell system (0.4-μm pore size). The coculture was maintained in SCM without the Schwann cell growth supplement. Six experimental groups were incubated for 48 h (37°C, 5% CO_2_): (1) THP-1 alone (Control); (2) THP-1 + 20 μM etifoxine (ETX); (3) THP-1 + HSC coculture; (4) coculture + 20 μM ETX; (5) coculture + 20 μM ETX + 2 μM RU486; and (6) coculture + 20 μM ETX + 20 μM GW9662. All compounds were dissolved in dimethyl sulfoxide (DMSO), and control groups received an equivalent volume of the solvent. Human Schwann cells (HSCs) were treated with 20 μM ETX for 48 h, after which the culture supernatant was collected and centrifuged to remove cell debris, yielding conditioned medium. This medium was subsequently used to treat macrophages for further validation experiments.

### Flow cytometry analysis

THP-1 monocytes were cultured and induced according to the experimental group, washed with PBS, enzymatically dissociated, and then washed twice with ice-cold PBS. For flow cytometry analysis, cells (1 × 10^6^/sample) were first incubated with an anti-CD16/32 antibody (1 μg/106 cells; BioLegend, #101303) in PBS/2% FBS for 15 min at 4°C to block Fc receptors. Surface staining was then performed with a PE-conjugated anti-CD206 antibody (1:50; BioLegend, #321105) in PBS/2% FBS for 30 min at 4°C in the dark. After two washes, the cells were fixed (eBioscience™ Fixation Buffer, #00-5523-00) for 15 min, permeabilized using the permeabilization buffer of the kit for 15 min, washed twice, and subsequently stained intracellularly with an APC-conjugated anti-CD68 antibody (BioLegend, #333810) in PBS/2% FBS for 30 min at 4°C in the dark. Following two final washes, the samples were acquired on a BD Accuri™ C6 Plus flow cytometer. Single cells were gated on FSC-A/FSC-H, and macrophage populations were quantified for CD68 and CD206 coexpression after fluorescence compensation using single-stained controls. The data were analyzed using FlowJo v10.8 software.

### Generation of TSPO-knockdown HSC stable cell line

To investigate the role of TSPO in ETX-induced progesterone production, we used a lentiviral shRNA system to generate a TSPO-knockdown HSC stable cell line. In preliminary experiments, we screened out the most effective shRNA sequence targeting human TSPO (5’-CGGCUCCUACCUGGUCUGGAATT-3’). This specific shRNA sequence was packaged into a lentiviral vector (PGMLV-Puro) by Genomeditech (Shanghai, China). According to the manufacturer’s instructions, the lentiviral particles were transduced into primary HSC cells. At 72 h post-infection, cells were selected with 8 μg/mL puromycin to eliminate non-transduced cells, thereby establishing a stable knockdown cell line. The knockdown efficiency of TSPO was validated by Western blot. The validated TSPO-knockdown HSC cells were expanded and used for subsequent experiments.

### Quantification of progesterone levels by ELISA

Primary human Schwann cells were treated with: (1) Ctrl (SCM; #1701); (2) 20 μM etifoxine in SCM (ETX); (3) 20 μM etifoxine in SCM following TSPO knockdown (shTSPO + ETX). THP-1 cells were treated in parallel with Ctrl or ETX medium. Cells were maintained at 37°C with 5% CO_2_ for 48 h, washed twice with ice-cold PBS, and lysed in RIPA buffer (50 μL per 1 × 10^7^ cells). The lysates were incubated on ice for 30 min (vortexed every 10 min) and centrifuged (12,000 × g, 15 min, 4°C), after which the supernatants were collected. The total protein concentration was quantified using a BCA assay. Progesterone (Pg) levels were measured using competitive ELISA (Elabscience #E-OSEL-H0011). Standards (78.13–5,000 pg/mL in SCM) and diluted lysates (50 μL/well) were loaded into antibody-coated wells, followed immediately by the addition of 50 μL of HRP-conjugated detection antibody (1:100). After an incubation for 60 min at 37°C, the plates were washed 5 × and incubated with 90 μL of TMB substrate (15 min, 37°C, dark), and the reactions were stopped with 50 μL of stop solution. The absorbance (450 nm) was measured, and the Pg concentrations were interpolated from a 4-parameter logistic (4-PL) standard curve.

### mRNA library construction

RNA was extracted from THP-1 cells using TRIzol (Invitrogen), quantified with a NanoDrop 2000 (Thermo Fisher Scientific), and mRNA was purified with oligo (dT) magnetic beads. cDNA libraries were constructed through random hexamer-primed RT–PCR, purified with Ampure XP beads, and subjected to quality control using an Agilent 2100 bioanalyzer. Double-stranded PCR products were denatured and circularized into ssCir DNA libraries. RNA-seq library construction and sequencing were performed by Oebiotech (Shanghai). Differentially expressed genes (DEGs) were identified with | log_2_(fold change)| > log_2_(1.2) (≈0.26) and adjusted *p* < 0.05. For the Kyoto Encyclopedia of Genes and Genomes (KEGG, RRID:SCR_012773) pathway analysis, a stringent threshold (| log_2_FC| > log_2_(1.5) ≈ 0.58) was applied. Gene Ontology (GO) analysis and gene set enrichment analysis (GSEA) were conducted using clusterProfiler (v4.0; RRID:SCR_016884).

### Western blot analysis

Cells were lysed in non-denaturing buffer (Applygen, #C1050) supplemented with 1% protease (#P6730) and phosphatase inhibitors (#P1260, Solarbio). The lysates were separated on AnyKD™ PAGE gels (Dakewe, #8012011/1071), transferred to PVDF membranes (Millipore, #IPVH00010), and blocked with 5% non-fat milk in TBST. The membranes were probed overnight at 4°C with the following primary antibodies: anti-PPARγ (CST, #2435), anti-CPT1A (CST, #12252), anti-PGC1α (CST, #2178), anti-BNIP3L (Selleck, F0469), anti-Parkin (Selleck, F0296), anti-NLRP3 (Selleck, F0335), anti-LC3B (Selleck, F0145), anti-p62 (Selleck, F0106), anti-GAPDH (CST, #97166), and anti-β-actin (Boao Ruijing; all 1:1,000 except β-actin, 1:5,000). After the membranes were incubated with HRP-conjugated secondary antibodies (anti-rabbit IgG, Abcam #ab6721; anti-mouse IgG, #ab205719; 1:5,000), the proteins were detected using ECL reagent (NCM Biotech, #P10300) on an Amersham Imager 600. The results of triplicate experiments were analyzed using ImageJ (RRID:SCR_003070) for grayscale quantification.

### Fatty acid oxidation colorimetric assay kit

The fatty acid oxidation (FAO) capacity was quantified using a colorimetric assay kit (Elabscience^®^ #E-BC-K784-M). Clarified supernatants from cell lysates (equivalent to 1 × 10^6^ cells) were prepared by centrifugation at 10,000 × g for 15 min at 4°C. Aliquots (50 μL) were loaded into 96-well plates, with a parallel setup of assay wells (containing chromogenic substrate) and control wells (buffer only). The following reagents were sequentially added: 20 μL of cofactor working solution, 145 μL of reaction mixture, and 20 μL of chromogenic agent. After a 30-min incubation at 37°C, the absorbance was measured at 450 nm. FAO activity (U/g protein) was calculated as follows: FAO = (Δ*A*450-*b*)/a÷30 × 1,000÷*C*pr × *f*, *where Cpr* = *total protein concentration (g/L), f* = *sample dilution factor, and 30* = *reaction time (min).*

### Monitoring of mitophagy using the mito-QC reporter system

A pH-sensitive fluorescent reporter system, mito-QC, was employed to monitor mitophagy. This system utilizes a mitochondrially targeted tandem mCherry-GFP tag, guided by a mitochondrial matrix targeting sequence (typically derived from cytochrome c oxidase subunit 8, Cox8), enabling its stable localization to mitochondria. The principle is based on the quenching of GFP fluorescence in the acidic environment of the autolysosome, while mCherry remains stable.

Stable polyclonal THP-1 cell lines expressing mito-QC were generated by lentiviral transduction with pLenti-MitoQC (WZ Biosciences Inc.) at an MOI of 25 in RPMI-1,640 medium containing 10% FBS for 72 h, followed by selection with 2 μg/mL puromycin for 7 days.

For the assay, cells were treated with experimental compounds or vehicle control for 48 h. Live-cell imaging was performed using a confocal microscope. EGFP and mCherry were excited at 488 nm and 561 nm, with emissions collected at 500–550 nm and 570–620 nm, respectively.

Quantification of mitophagy was performed based on the fluorescence pattern: intact mitochondria appeared as yellow structures (colocalization of EGFP and mCherry), while mitophagic events were identified as distinct red puncta (positive for mCherry but negative for EGFP). The level of mitophagy was quantified by counting the number of discrete red puncta (mCherry-only puncta) per cell. These red puncta indicate that the mitochondria have been delivered to acidic autolysosomes, a process typically associated with colocalization of the autophagosome marker protein LC3, serving as key evidence for mitochondrial degradation via the autophagy pathway.

### Establishment of an animal model of sciatic nerve crush injury

#### Animals

Female Sprague-Dawley (SD) rats aged 8 weeks, weighing 200–250 g, were used in this study and obtained from GemPharmatech LLC. All animals were housed under specific pathogen-free (SPF) conditions, with the room temperature maintained at 22 ± 2°C and humidity at 50 ± 10%. A 12-h light/dark cycle was applied, and the rats had free access to food and water. All animal experimental protocols were approved by the Institutional Animal Care and Use Committee (IACUC) of Novel Science (Beijing) Co., Ltd. (Ethical Approval No. NWAKLL-2025-03-06) and were reported in accordance with the ARRIVE guidelines.

#### Surgery and postoperative analgesia

Surgeries were performed under aseptic conditions. Rats were anesthetized with isoflurane inhalation (4% for induction, 2% for maintenance) and fixed in a prone position. A 5 mm incision was made distal to the right sciatic tuberosity, and the sciatic nerve was exposed by separating the biceps femoris muscle. The nerve was compressed with maximum force using non-toothed forceps for 10 s per compression, repeated for three cycles, to induce nerve fascicle separation. The injury site was marked with a 10–0 suture. Subsequently, the muscle and skin incisions were closed with 4–0 nylon sutures. To minimize postoperative pain, all rats received subcutaneous injections of buprenorphine (0.05 mg/kg, every 12 h for 3 consecutive days) preoperatively and postoperatively.

#### Animal grouping and drug administration

Post-surgery, rats were randomly divided into four groups (*n* = 6 per group, with 3 rats per time point for behavioral and histological analyses on postoperative days 3, 7, and 15). From the day of surgery until the experimental endpoint, rats in each group received daily intraperitoneal injections: the control group received an equal volume of the solvent; the ETX group received etifoxine (50 mg/kg); the ETX + GW9662 group received etifoxine (50 mg/kg) combined with GW9662 (1 mg/kg); and the ETX + RU486 group received etifoxine (50 mg/kg) combined with RU486 (20 mg/kg). All drugs were dissolved in DMSO, and the injection volumes were kept consistent.

### Assessment of the mechanical pain threshold

An electronic pressure algometer was used on the bilateral hindpaw mid-plantar surface to assess the mechanical paw withdrawal threshold (PWT). Measurements were conducted on postintervention days 0, 3, 7, and 15. Pressure was applied vertically to the central plantar surface of the rat hindpaw until the paw withdrawal reflex was elicited; the force value at withdrawal was recorded (upper limit 10 N). Three trials per paw (intervals ≥ 5 min) were performed, and the mean value per paw was calculated; then, the mean values of the bilateral paws were averaged again to yield the final PWT.

### Assessment of hindlimb grip strength

Hindlimb grip strength (HGS) was measured using a T-bar meter. The rats underwent three 5-min daily acclimation sessions. During testing, restrained rats were subjected to horizontal tail traction perpendicular to the T-bar until paw disengagement; the peak prefailure force (N) was recorded. Three trials (≥ 5-min intervals) per rat were averaged and normalized to body weight (g), expressed as N/g. *n* = 3 animals per group per time point.

### Immunofluorescence staining

Sections (5 μm) of formalin-fixed, paraffin-embedded sciatic nerves were subjected to antigen retrieval in citrate buffer (pH 6.0, 95°C, 15 min), followed by deparaffinization, permeabilization with 0.3% Triton X-100 (30 min, room temperature), and blocking with 5% BSA (1 h, room temperature). The sections were incubated overnight at 4°C with the following primary antibodies: mouse anti-CD68 (1:200; Santa Cruz sc-20060), rabbit anti-CD206 (1:500; CST #24595), rabbit anti-NF200 (1:500; CST #30564), and rabbit anti-PPARγ (1:300; e.g., CST #2435). Then, the sections were incubated for 2 h at room temperature in the dark with the corresponding secondary antibodies: Alexa Fluor 488-conjugated goat anti-rabbit IgG and Alexa Fluor 594-conjugated goat anti-mouse IgG. Nuclei were counterstained with DAPI, and slides were mounted with ProLong Gold Antifade Mountant. In the quantitative analysis of immunofluorescence, three non-serial sections were selected from each animal, and images were captured using 5 ×, 40 ×, and 63 × objective lenses. ImageJ software was employed to count colocalized or positively stained cells. The proportion of M2-type macrophages was assessed by calculating the percentage of cells showing colocalization of CD68 and CD206 relative to the total number of CD68-positive cells, using the formula: (number of CD68 + CD206 + cells/total number of CD68 + cells) × 100%. The percentage of NF200-positive area was determined by measuring the ratio of the NF200-positive region to the total visual field area. Quantitative data for each group were obtained from three animals.

### Retrograde tracing and labeling

To assess axonal regeneration of motor neurons, on postoperative day 12 (for rats designated for sacrifice on postoperative day 15), the gastrocnemius branch of the sciatic nerve was re-exposed under anesthesia. Using a Hamilton microsyringe, 1 μL of 1% CTB555 (Cholera Toxin Subunit B conjugated to Alexa Fluor 555; Invitrogen #C22843) was slowly injected at a rate of 0.2 μL/min. The needle was left in place for 5 min after injection. After allowing 3 days for retrograde transport of the tracer (i.e., on postoperative day 15), rats were deeply anesthetized (sodium pentobarbital, 50 mg/kg, intraperitoneal injection) and transcardially perfused with 4% paraformaldehyde. The lumbosacral enlargement (L4–S1) of the spinal cord was then removed and serially sectioned on a cryostat at a thickness of 40 μm in the sagittal plane. Using systematic sampling, one section out of every six (240 μm intervals) was selected. Under a fluorescence microscope (excitation wavelength 561 nm), all CTB555-positive neurons with intact cell bodies and clearly visible nucleoli were counted. The results were expressed as the average number of positive neurons per section.

### Statistics

All the statistical values were calculated with GraphPad Prism 9.0 (GraphPad Prism, RRID:SCR_002798). One-way ANOVA was used for comparisons between more than two groups, and Tukey’s multiple comparisons test was used for multiple comparisons. The quantitative data are presented as the means ± standard deviations (SDs) or the means ± standard errors of the means (SEMs) from at least three samples or experiments per data point. A *p* < 0.05 was considered to indicate statistical significance.

## Results

### Etifoxine promotes M2 macrophage polarization via Schwann cell-dependent mechanisms

**FIGURE 1:**
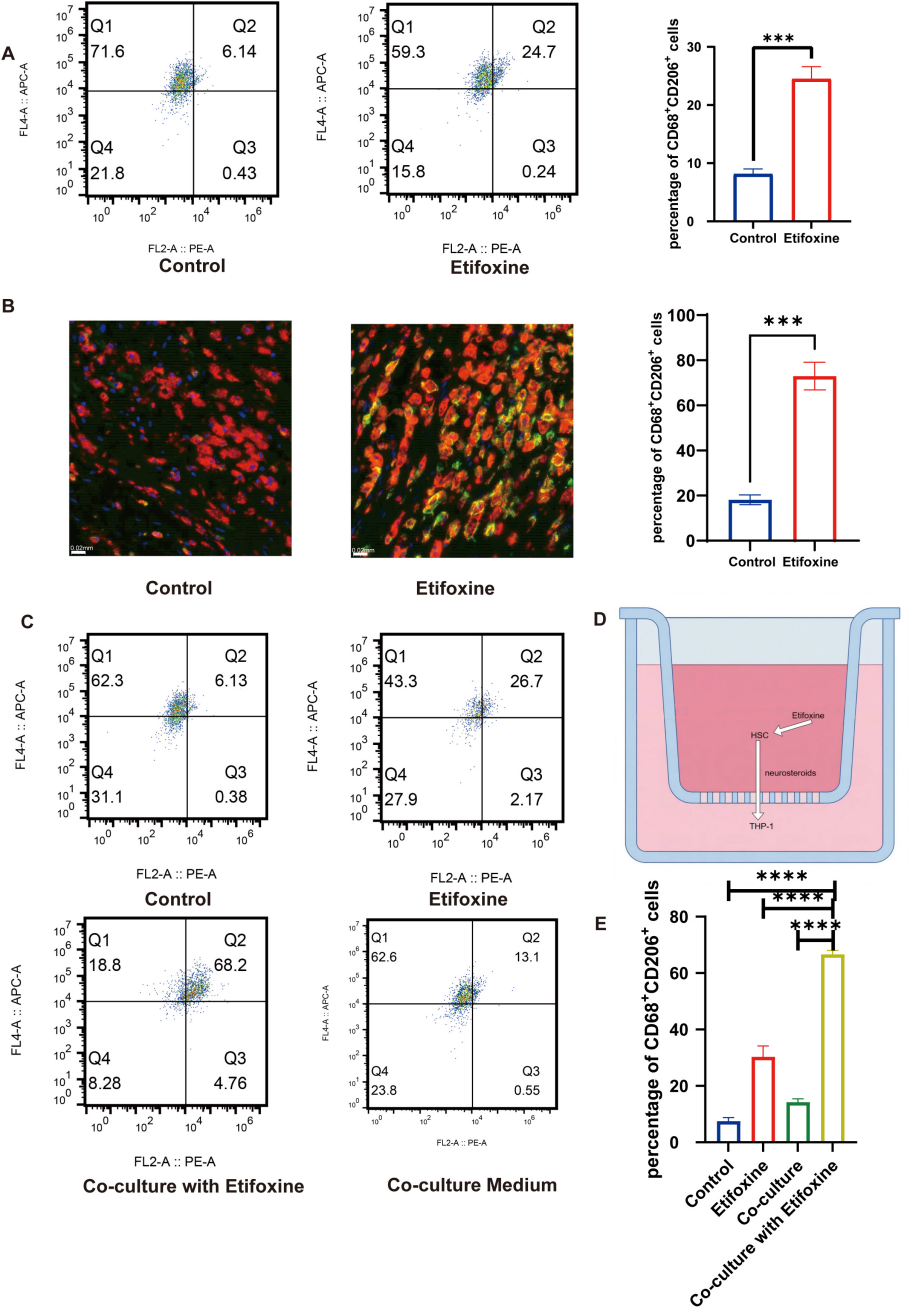
ETX promotes M2 macrophage polarization through Schwann cell-dependent mechanisms. **(A)** Left panel: Flow cytometry plots of CD68-APC and CD206-PE. Right panel: Quantification of the CD68^+^CD206^+^ population. The data are presented as means ± SEMs; *n* = 3; ***P < 0.0001 compared with the Ctrl (Student’s *t-*test). **(B)** Immunofluorescence staining for CD68 (red) and CD206 (green) at 3 days post-injury. Scale bar: 50 μm. **(C)** Flow cytometry plots of cells cultured under four conditions: i. Ctrl (macrophages alone); ii. ETX (macrophages + ETX); iii. coculture (HSCs + macrophages + ETX); iv. coculture medium. **(D)** Experimental schematic of the Schwann cell (HSC)–macrophage (THP-1) coculture system. **(E)** Quantification of CD68^+^CD206^+^ cells. The data are presented as means ± SEMs; *n* = 3; ****P < 0.0001 (one-way ANOVA, Tukey’s test).

We investigated the regulatory effect of ETX on macrophage polarization. *In vitro* experiments showed that, compared with the untreated group, ETX treatment increased the proportion of CD68^+^CD206^+^ cells in THP-1 cells—a phenotype marking M2 polarization (Figure 1A; mean ± SEM; *n* = 3; *P* < 0.001; Student’s *t*-test). This M2-promoting effect was also confirmed *in vivo*. Compared with the control group, ETX-treated rats with sciatic nerve crush injury exhibited enhanced M2 polarization on day 3 post-injury, as supported by immunofluorescence co-localization of CD68 (red) and CD206 (green) (Figure 1B). Notably, although ETX promoted M2 macrophage polarization both *in vitro* and *in vivo*, its effect was markedly stronger *in vivo*. This difference may stem from the unique *in vivo* microenvironment, where complex intercellular interactions further shape the polarization state of macrophages.

We hypothesized that the stronger M2-polarizing effect of ETX observed *in vivo* may be related to intercellular interactions within the local microenvironment. To test this hypothesis, we established a coculture system of human Schwann cells (HSCs) and THP-1 macrophages to mimic the cellular interactions occurring in the nerve injury area *in vivo*. As macrophages themselves lack the key enzymes required for neurosteroid synthesis, the precise mechanism by which they are induced toward an M2 phenotype in peripheral nerve injury has yet to be fully elucidated (Figure 1D). Flow cytometry revealed significant differences across treatment conditions (Figures 1C,E). Coculture alone (HSCs + macrophages) or ETX treatment alone (macrophages + ETX) induced only modest M2 polarization relative to the macrophage-only control (Ctrl). In contrast, ETX-treated cocultures (HSCs + macrophages + ETX) exhibited a substantial increase in the CD68^+^CD206^+^ population. Statistical analysis confirmed significant intergroup differences (means ± SEMs; *n* = 3; *P* < 0.05; one-way ANOVA with Tukey’s *post-hoc* test).

Together, these findings demonstrate that ETX induces M2 macrophage polarization, and this effect is potently enhanced in the presence of Schwann cells, suggesting that soluble factors derived from Schwann cells contribute to ETX-mediated M2 polarization.

### ETX promotes M2 polarization via progesterone-mediated activation

**FIGURE 2:**
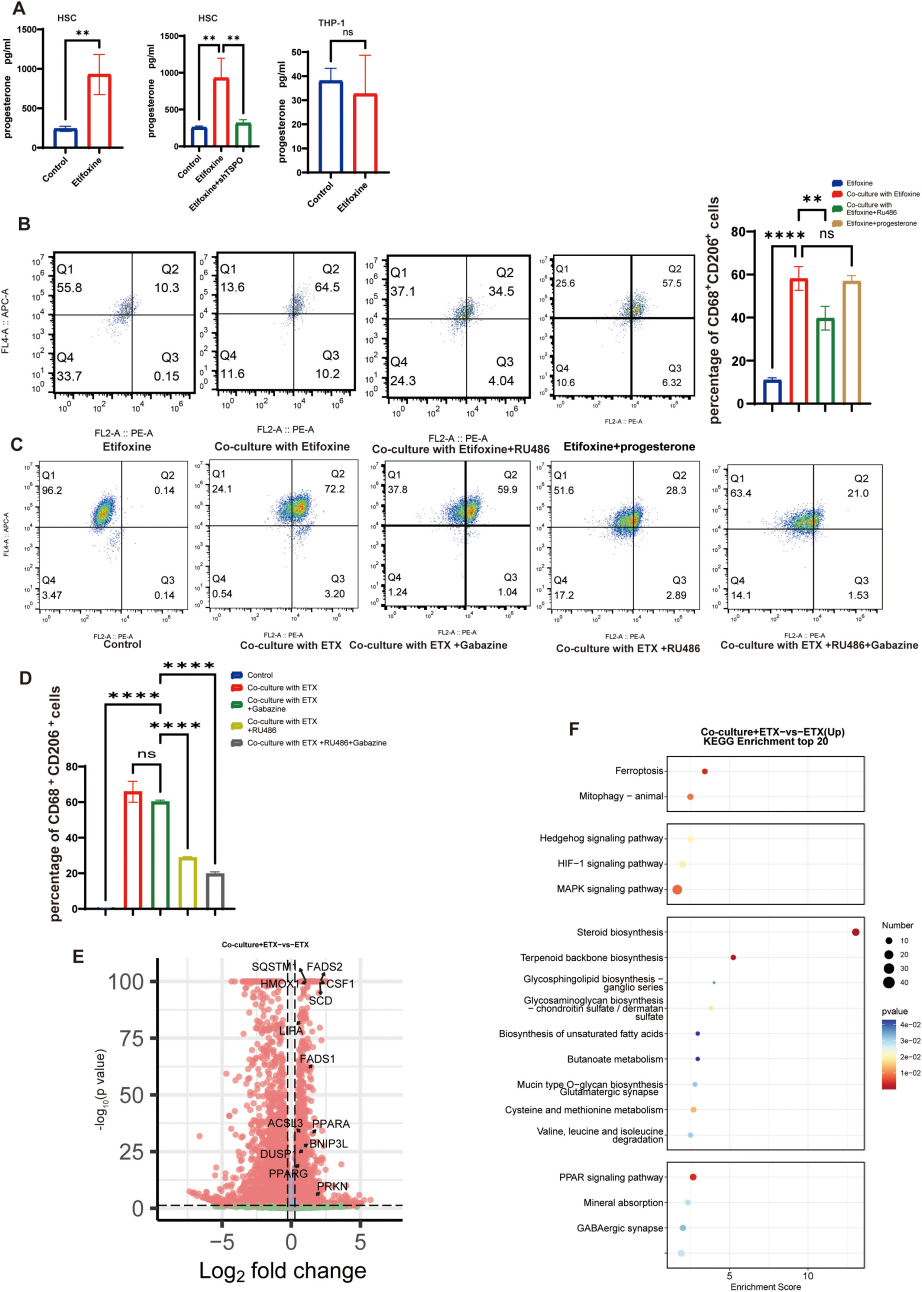
ETX Promotes M2 polarization via progesterone-mediated activation. **(A)** Left panel: Progesterone (Pg) levels are increased in ETX-treated HSCs (48 h). Middle panel: TSPO knockdown blocks ETX-dependent Pg production in HSCs. Right panel: ETX treatment fails to upregulate Pg levels in THP-1 cells. Data are presented as means ± SEMs; *n* = 3; **P < 0.01 compared with the Ctrl (left panel, unpaired *t*-test); **P < 0.01 compared with ETX, ns, not significant (middle and right panels, one-way ANOVA, with Tukey’s *post-hoc* test). **(B)** Flow cytometry analysis of CD68^+^CD206^+^ cells in cocultures. i. Ctrl (Etifoxine); ii. cocultures + ETX; iii. cocultures + ETX + RU486 (RU486; 2 μM). iv. ETX + Progesterone. Data are presented as means ± SEMs; *n* = 3; **P < 0.01, ****P < 0.0001, ns, not significant (one-way ANOVA with Tukey’s *post-hoc* test). **(C)** Flow cytometry analysis of CD68 + CD206 + cells in co-cultures. i. Control; ii. Co-culture with ETX; iii. Co-culture with ETX + Gabazine; iv. Co-culture with ETX + RU486; v. Co-culture with ETX + RU486 + Gabazine. **(D)** Quantitative analysis of CD68 + CD206 + cells from the flow cytometry data shown in **(C)**. Data are presented as means ± SEMs; *n* = 3; ****P < 0.0001, ns, not significant (one-way ANOVA with Tukey’s *post-hoc* test). **(E)** RNA-seq analysis of DEGs (ETX coculture vs. ETX alone). Volcano plot: log_2_FC > 0.263 (1.2-fold change), *p* < 0.05. **(F)** KEGG enrichment analysis of upregulated DEGs. The top pathways are shown (bubble size: gene ratio; color: −log10(*P*); threshold: log2FC > 0.585).

ETX enhanced the synthesis of progesterone (Pg) in human Schwann cells. Compared with the untreated control group, progesterone levels were significantly increased in ETX-treated human Schwann cells at 48 h (Figure 2A; mean ± SEM; *n* = 3; *P* < 0.01; unpaired *t*-test). In contrast, THP-1 macrophages did not effectively secrete progesterone under either basal conditions or ETX stimulation (Figure 2A). In TSPO-knockdown human Schwann cells (Supplementary Figure 1E), the ETX-induced progesterone synthesis effect was abolished (mean ± SEM; *n* = 3; compared with ETX treatment alone, *P* < 0.01; one-way ANOVA followed by Tukey’s test). In the human Schwann cell-THP-1 macrophage co-culture system, ETX treatment significantly increased the proportion of M2-type macrophages. Importantly, this pro-M2 polarization effect was significantly attenuated by the potent progesterone receptor antagonist RU486 (2 μM). Exogenous progesterone supplementation further enhanced M2 polarization in the group treated with ETX alone (Figure 2B), and treating macrophages with progesterone alone also significantly promoted their M2 polarization (Supplementary Figure 1C). To further confirm that factors secreted by Schwann cells are sufficient to drive this phenotype, treatment of macrophages with conditioned medium from ETX-pretreated Schwann cells similarly significantly promoted M2 polarization (Supplementary Figure 1D). These results collectively indicate that progesterone synthesized and secreted by Schwann cells upon ETX stimulation plays a key role in promoting macrophage M2 polarization. However, the observation that the progesterone receptor antagonist RU486 only partially attenuated this effect suggests that additional, complementary mechanisms may also contribute.

Given that ETX is not only a ligand for TSPO but also an agonist for the β2/3 subunits of the GABAA receptor (Barresi et al., 2021), to confirm whether its pro-M2 polarization effect is specifically dependent on TSPO activation, we validated this using the TSPO-selective ligand 4’-chlorodiazepam (Ro5-4864). Treatment with Ro5-4864 (30 μM) yielded results consistent with those observed for ETX (Supplementary Figures 1A,B). To further corroborate our findings, we added the GABAA receptor antagonist Gabazine to the ETX-treated co-culture system to block GABA signaling. The results showed that Gabazine (10 μM) only partially attenuated M2 polarization, an effect that was less pronounced than that of RU486. This suggests that while GABAergic signaling may play a minor or indirect role in this *in vitro* coculture system, the progesterone receptor-mediated pathway is the dominant driver of ETX-induced M2 polarization. Dual blockade using both RU486 and Gabazine further supported this observation (Figures 2C,D). To delve deeper into the neurosteroid-mediated mechanisms downstream of progesterone receptor activation, we moved beyond the effects of ETX treatment alone and performed RNA sequencing on macrophages from different treatment groups. Compared to macrophages treated with ETX alone, macrophages from the ETX-treated co-culture system exhibited a distinct transcriptional profile. Volcano plot analysis revealed significant upregulation of several key genes, including PPARG, BNIP3L, CSF1, DUSP1, and PRKN (*P* < 0.05) (Figure 2E). KEGG enrichment analysis of the differentially expressed genes indicated significant enrichment in the PPAR signaling pathway, mitophagy, steroid biosynthesis, and the MAPK signaling pathway (Figure 2F).

### Schwann cell-derived progesterone activates the PPARγ–PGC1α axis to reprogram fatty acid oxidation in macrophages

**FIGURE 3:**
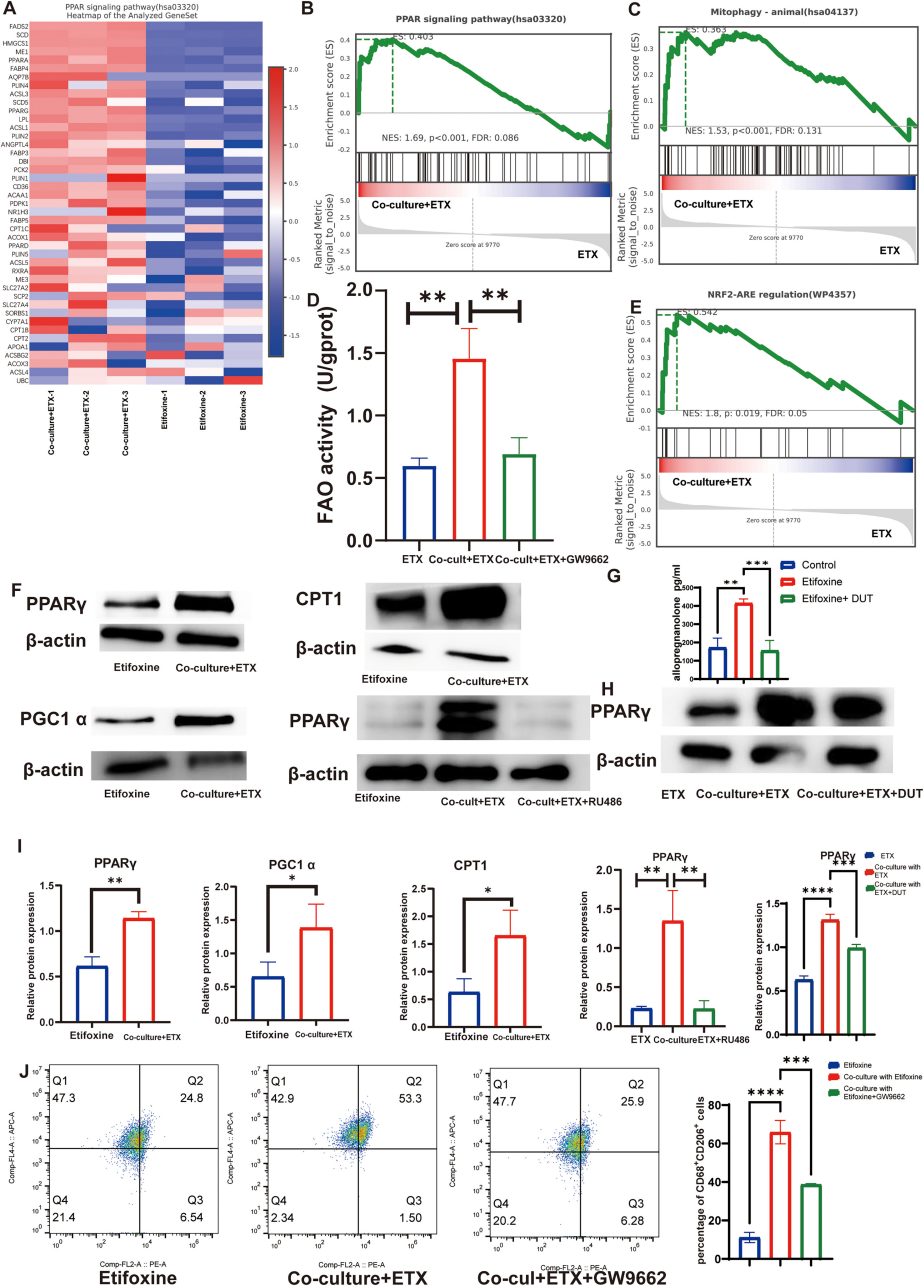
Progesterone drives PPARγ-mediated fatty acid oxidation and mitophagy activation. **(A)** Expression of genes in the PPAR signaling pathway. The heatmap shows the RNA-seq data (rows: z scores; columns: samples). **(B)** GSEA of PPAR signaling. ETX coculture vs. ETX alone (NES = 1.69, FDR = 0.086). **(C)** GSEA of mitophagy. ETX coculture vs. ETX alone (NES = 1.53, FDR = 0.131). **(D)** Fatty acid oxidation (FAO) capacity in co-cultures. The levels of FAO were measured by ELISA in ETX-treated HSCs, co-cultures with ETX, and co-cultures with ETX plus GW9662. Data are presented as means ± SEMs; *n* = 3; **P < 0.01 (one-way ANOVA followed by Tukey’s test). **(E)** GSEA of NRF2-ARE signaling (NES = 1.8, FDR = 0.05). **(F)** Western blots showing the levels of PPARγ, CPT1A, and PGC1α in cocultures ± ETX/RU486 (RU486, 2 μM). **(G)** Allopregnanolone levels in different groups. The concentrations of allopregnanolone were measured by ELISA in control, ETX-treated, and ETX with Dutasteride groups. Data are presented as means ± SEMs; *n* = 3; **P < 0.01, ***P < 0.001 (one-way ANOVA followed by Tukey’s test). **(H)** Western blots showing the levels of PPARγ. **(I)** Densitometry of the blots in **(F)**. The data are presented as means ± SEMs; *n* = 3; *P < 0.05, **P < 0.01, ***P < 0.001, ****P < 0.0001 (Student’s *t-*test, one-way ANOVA followed by Tukey’s test). **(J)** Flow cytometry analysis of CD68^+^CD206^+^ cells in cocultures. i. Ctrl (Etifoxine); ii. cocultures + ETX; iii. cocultures + ETX + GW9662 (GW9662; 10 μM). Data are presented as means ± SEMs; *n* = 3; ***P < 0.001, ****P < 0.0001 (one-way ANOVA followed by Tukey’s test).

In this study, RNA-seq analysis revealed significant activation of PPARG in the ETX-treated co-culture system (Figure 2C). Gene set enrichment analysis (GSEA) further confirmed the enrichment of the PPAR signaling pathway (NES = 1.69, FDR = 0.086; Figure 3B). Heatmap results showed coordinated upregulation of key PPAR pathway-related genes (PPARG, PPARA, and CPT1B) in the ETX-treated co-culture group (Figure 3A), suggesting that activation of the PPAR pathway is dependent on the presence of Schwann cells. Compared to macrophages treated with ETX alone, pathway activation was observed exclusively under co-culture conditions, further supporting its dependence on intercellular crosstalk.

Given the established role of the PPARγ–PGC1α axis in regulating fatty acid oxidation (FAO) and driving macrophage M2 polarization, this study further explored the functional significance of this pathway in THP-1 macrophages. The results demonstrated that ETX treatment significantly enhanced FAO capacity in the co-culture system, and this effect was completely abolished by the PPARγ-specific antagonist GW9662 (Figure 3D; *P* < 0.01), indicating that the enhancement of FAO is dependent on PPARγ activation. Western blot analysis further confirmed that protein levels of PPARγ, its transcriptional coactivator PGC1α, and the key FAO rate-limiting enzyme CPT1A were significantly increased in the ETX-treated co-culture group (Figure 3F).

To identify the upstream regulatory signals, we examined the roles of progesterone and its metabolites. The results showed that the progesterone receptor antagonist RU486 (2 μM) inhibited the upregulation of PPARγ (*P* < 0.01; Figures 3F,I), suggesting that progesterone signaling is a upstream event in this pathway. The progesterone metabolite allopregnanolone has also been reported to activate PPARγ, ELISA assays confirmed that ETX treatment indeed promoted allopregnanolone production (Figure 3G). To further distinguish the contributions of progesterone itself versus its metabolite, we used the SRD5A1 inhibitor dutasteride (10 μM) to block the conversion of progesterone to allopregnanolone. This intervention resulted in only a partial reduction in both allopregnanolone levels and PPARγ expression, with the inhibitory effect being significantly less pronounced than that observed with RU486 treatment (Figures 3G,H). This indicates that progesterone itself, acting through the progesterone receptor to activate PPARγ, is the dominant mechanism in this regulatory axis, although a contributory role for its metabolite, allopregnanolone, cannot be entirely excluded.

Finally, to validate the functional contribution of PPARγ activation to the M2 polarization phenotype, we assessed M2 marker expression following pathway inhibition. The results demonstrated that the enhanced M2 polarization induced by ETX co-culture was abrogated upon GW9662 treatment (Figure 3J), demonstrating that PPARγ activation is required for progesterone-driven macrophage M2 polarization in this coculture system.

### PPARγ activation further induces mitophagy and sustains fatty acid oxidation.

**FIGURE 4:**
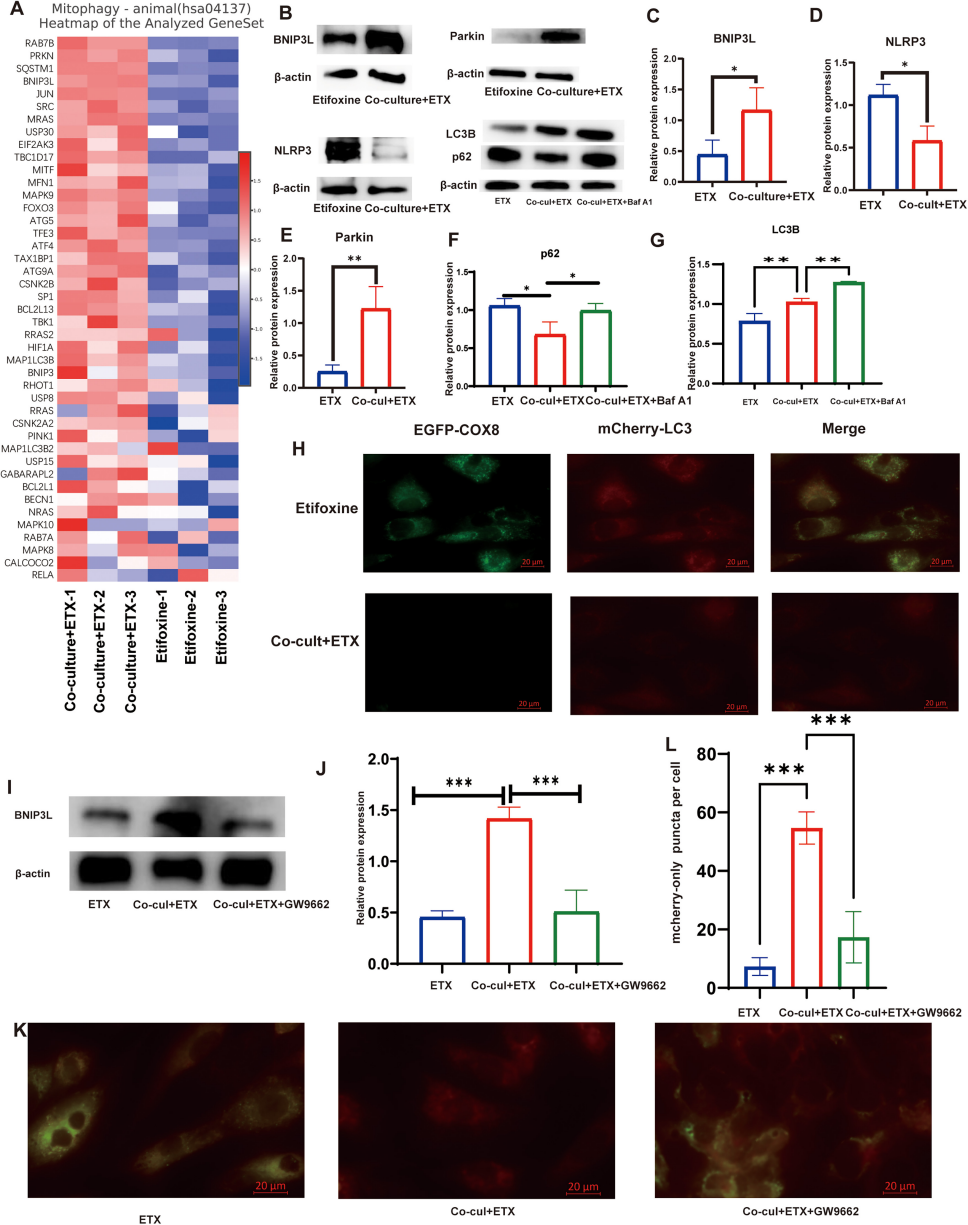
Progesterone drives mitophagy via PPARγ-dependent signaling. **(A)** Mitophagy-related gene expression. The heatmap shows the RNA-seq data (rows: z scores; columns: samples). **(B)** Top panel: Western blot analysis of autophagy flux. The levels of mitophagy markers in cocultures treated with ETX ± bafilomycin A1 (Baf A1, 50 nM; lysosomal inhibitor) are shown. Bottom panel: loading control (β-actin). **(C–G)** Quantification of the protein levels in **(B)**: BNIP3L, Parkin, NLRP3, LC3-II, and p62. Data are shown as means ± SEMs; *n* = 3; *P < *0.05* and **P < 0.01 compared with ETX alone (Student’s *t*-test, one-way ANOVA with Tukey’s *post-hoc* test). **(H)** Mitophagy flux was measured using the mito-QC reporter. Representative confocal images show mCherry^+^GFP^–^ puncta (red) that indicate mitochondria in lysosomes; the GFP signal (green) is quenched in acidic compartments. Scale bar: 20 μm. **(I)** Top panel: BNIP3L expression after PPARγ inhibition. WB of cocultures treated with ETX ± GW9662 (10 μM) is shown. Bottom panel: β-actin control. **(J)** Quantification of the BNIP3L levels in **(I)**. Data are presented as means ± SEMs; *n* = 3; ***P < 0.01 compared with ETX (one-way ANOVA with Tukey’s *post-hoc* test). **(K)** PPARγ-dependent mitophagy. Representative images show the levels of the mito-QC reporter in ETX ± GW9662-treated macrophages. Scale bar: 20 μm. **(L)** Statistical analysis was performed using one-way ANOVA to compare the number of mCherry-only puncta per cell among the three groups: ETX, coculture + ETX, and coculture + ETX + GW9662. ***P < 0.001 (one-way ANOVA with Tukey’s *post-hoc* test).

Volcano plots of RNA-seq data revealed significant upregulation of genes related to mitophagy. Subsequent Gene Set Enrichment Analysis (GSEA) confirmed the concurrent activation of both mitophagy and the NRF2 signaling pathway (Figures 3C,E). Further examination of mitophagy-related genes indicated that ETX-treated co-cultures exhibited a significant alteration in the expression profile of this pathway (Figure 4A), suggesting that PPARγ may induce mitophagy to maintain metabolic homeostasis following the activation of fatty acid oxidation. Western blot analysis showed that ETX-treated co-cultures displayed significantly increased protein levels of key mitophagy markers (BNIP3L and Parkin) and the autophagosome-associated protein LC3-II, alongside decreased expression of the autophagy substrate p62 and the inflammasome component NLRP3. Co-treatment with the lysosomal inhibitor Bafilomycin A1 (Baf A1) further exacerbated LC3-II accumulation and p62 aggregation, confirming enhanced autophagic flux (Figures 4B–G). Visualization using the mito-QC reporter system demonstrated a significant increase in the number of mCherry-only puncta (representing mitochondria within acidic lysosomes, i.e., mitolysosomes) in the ETX-treated co-culture group (Figure 4H) and significantly suppressed the ETX-induced increase in mCherry-only puncta detected by the mito-QC system (Figure 4K). Collectively, these findings indicate that PPARγ activation induces mitophagy to sustain fatty acid oxidation.

### ETX Promotes multidimensional nerve repair after sciatic nerve crush injury through macrophage polarization and enhanced axonal regeneration

**FIGURE 5:**
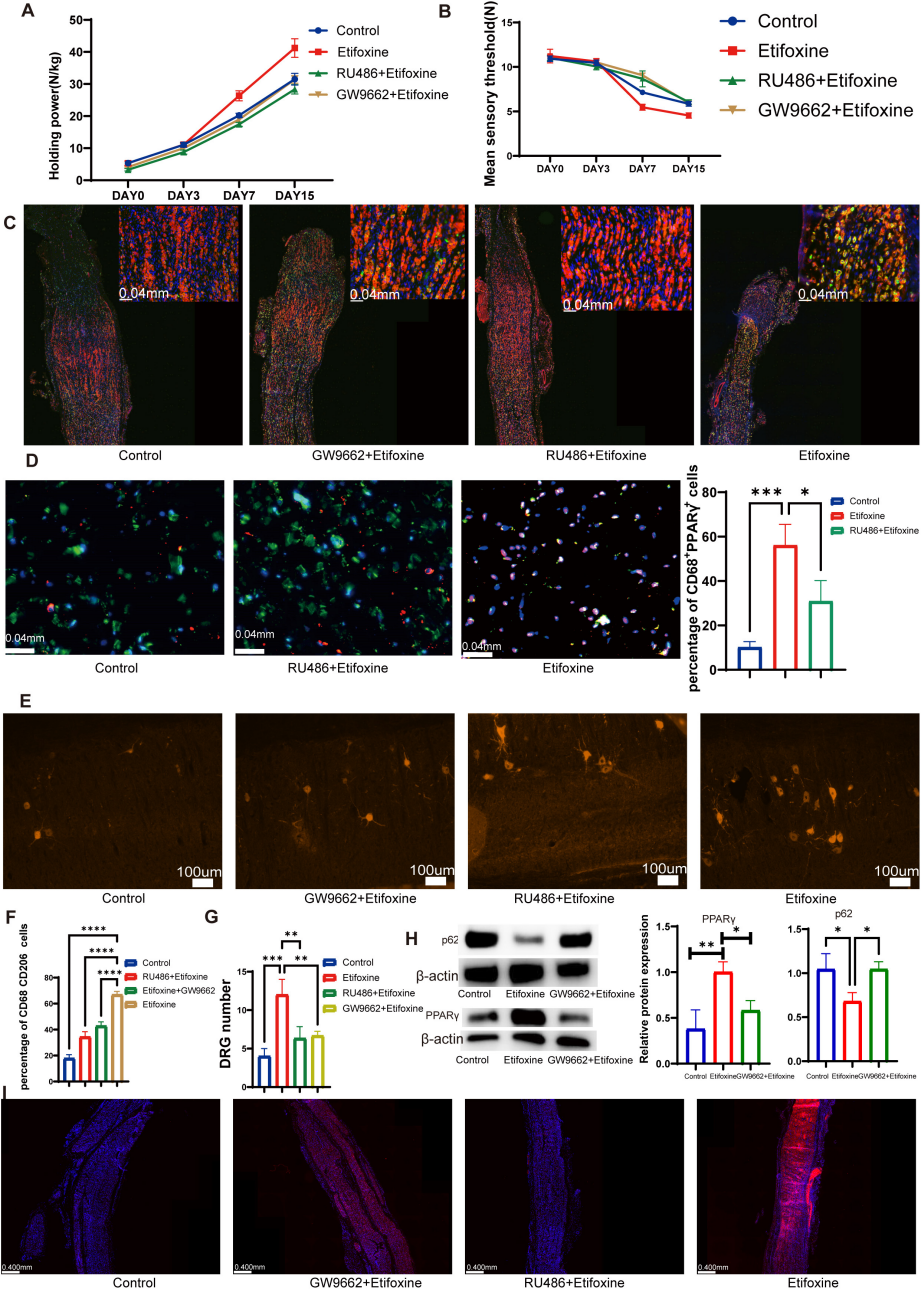
ETX promotes functional recovery and nerve repair following sciatic nerve crush injury in rats. **(A)** Hindlimb grip strength of ETX-treated and control rats at the indicated time points post-injury. Data are presented as the means ± SEMs (*n* = 3 per group). Statistical significance (*P* < 0.05) was determined by two-way ANOVA. **(B)** Mechanical pain threshold (g) of the hindpaws of the ETX-treated and control rats. Data are presented as the means ± SEMs (n = 3 per group). Statistical significance (*P* < 0.05) was determined by two-way ANOVA. **(C)** Representative images for M2 macrophages (CD68^+^CD206^+^) in injured sciatic nerves at 7 days post-injury among four groups: Control, Etifoxine, Etifoxine + RU486, and Etifoxine + GW9662. CD68 was labeled in red and CD206 in green. Scale bar: 0.04 mm. **(D)** Immunofluorescence staining and of macrophages (CD68^+^PPARγ^+^) in injured sciatic nerves at 7 days post-injury among three groups: Control, Etifoxine, and Etifoxine + RU486. CD68 was labeled in green and PPARγ in red. Scale bar: 0.04 mm. The percentage of CD68^+^PPARγ^+^ cells is presented as the means ± SEM; *n* = 3 per group; *P < 0.05, ***P < 0.001 (one-way ANOVA with Tukey’s *post-hoc* test). **(E)** Retrograde tracing of regenerating neurons in the spinal cord at 15 days post-injury. Scale bar: 100 μm. **(F)** The percentage of CD68^+^CD206^+^ double-positive cells is presented as the mean ± SEM (*n* = 3 per group). ****P < 0.0001 (one-way ANOVA with Tukey’s *post-hoc* test). **(G)** The number of retrogradely labeled neurons is presented as the mean ± SEM (*n* = 3 per group). **P < 0.01, ***P < 0.001 (one-way ANOVA with Tukey’s *post-hoc* test). **(H)** Western blot analysis of PPARγ and p62 proteins in the local injured tissue on day 7 after sciatic nerve injury. Representative Western blot bands and quantitative statistical graphs of PPARγ and p62 protein levels from each group. Data are presented as means SEMs; n = 3 per group; *P < 0.05, **P < 0.01 (one-way ANOVA with Tukey’s *post-hoc* test). **(I)** Immunofluorescence staining of regenerating axons (NF200^+^) in injured sciatic nerves at 7 days post-injury among four groups: Control, Etifoxine, Etifoxine + RU486 and Etifoxine + GW9662. NF200 was labeled in red.

Administration of ETX significantly promoted both functional and structural recovery after sciatic nerve crush injury in rats. Two-way ANOVA indicated that ETX treatment had a significant main effect on functional recovery. Compared with the control group, ETX-treated rats showed sustained and significant improvements in hindlimb grasping force and mechanical pain threshold at multiple time points after injury (Figures 5A,B), as confirmed by *post-hoc* analysis at each time point (detailed behavioral data are provided in Supplementary File 1).

Immunofluorescence analysis on day 7 post-injury revealed that ETX treatment effectively increased the infiltration of pro-regenerative M2-type macrophages (CD68^+^CD206^+^) within the injured nerve. This effect could be attenuated by co-administration of RU486 or GW9662 (Figure 5C). Quantitative analysis confirmed that the percentage of CD68^+^CD206^+^ double-positive cells in the ETX group was significantly higher than in all other groups (Figure 5F). Furthermore, ETX significantly upregulated the co-expression of CD68 and PPARγ in macrophages, an effect that was partially reversed by RU486 (Figure 5D).

Concomitant with these immunophenotypic changes, we further investigated the underlying molecular mechanisms. Western blot analysis of the injury site on day 7 post-injury showed that ETX treatment significantly upregulated PPARγ protein levels, while p62 was decreased (Figure 5H), consistent with enhanced autophagic flux observed *in vitro*. This upregulation was consistent with the observed increase in PPARγ^+^ macrophages (Figure 5D), suggesting activation of the PPARγ signaling pathway, which is known to be associated with enhanced cellular repair processes.

Alongside the molecular alterations, axonal regeneration was substantially promoted. Retrograde tracing on day 15 post-injury demonstrated that the number of labeled regenerated neurons in the spinal cord was significantly greater in ETX-treated rats than in the control group (Figures 5E,G). Consistent with this, immunofluorescence staining of tissues on day 15 post-injury indicated that NF200^+^ axonal regeneration was more extensive in the ETX group compared to the control and inhibitor combination groups (Figure 5I).

In summary, these results demonstrate that ETX promotes functional recovery and multifaceted neural repair by enhancing M2 macrophage polarization, activating the PPARγ signaling pathway, and accelerating axonal regeneration.

## Discussion

This study reveals a mechanism by which the TSPO ligand etifoxine (ETX) promotes peripheral nerve repair via Schwann cell–macrophage metabolic reprograming. Our core findings indicate that (1) ETX activates TSPO in Schwann cells, inducing the synthesis of neurosteroids such as progesterone; (2) Pg subsequently activates the PPARγ–PGC1α signaling axis in macrophages through paracrine signaling, driving metabolic reprograming toward fatty acid oxidation (FAO); (3) PPARγ synchronously induces BNIP3L-mediated mitophagy to maintain FAO homeostasis; and (4) this cascade collaboratively promotes the M2 polarization of macrophages, ultimately accelerating the structural and functional recovery of peripheral nerves. These findings provide insights into the cellular and molecular mechanisms underlying ETX-induced nerve regeneration.

Some divergence in previous research regarding the roles of TSPO and its ligands in macrophage polarization has been noted: TSPO expression is significantly increased in perihematomal activated microglia/macrophages (including both M1 and M2 types), but cellular experiments (using pharmacological activation and genetic knockout) have demonstrated that TSPO is a negative regulator of the release of the proinflammatory factors TNF-α and IL-6 (Bonsack et al., 2016). TSPO is highly expressed in synovial macrophages in osteoarthritis. TSPO overexpression promotes M1 macrophage polarization, while its ligand Emapunil effectively inhibits M1 macrophage polarization (Yin et al., 2025). Notably, in our study, although THP-1-derived macrophages themselves could not effectively secrete progesterone (Pg) and ETX failed to stimulate Pg production in these cells, ETX monotherapy did, to some extent, increase the proportion of M2-polarized macrophages. This observation suggests an additional, direct mechanism: TSPO ligands such as ETX may also act by directly binding to mitochondrial TSPO on macrophages, thereby inhibiting the activation of the key pro-inflammatory NF-κB signaling pathway within these cells (Monga et al., 2019). This direct action would suppress the production of inflammatory cytokines and facilitate a phenotypic switch from the aggressive M1 state to the reparative M2 state, contributing to the overall anti-inflammatory and pro-repair effects. Given that TSPO facilitates cholesterol transport into the mitochondrial matrix to initiate neurosteroid synthesis (Rupprecht et al., 2022), we explored its role in this peripheral nerve injury model. Using a Transwell coculture model, we showed that TSPO agonists function via the “Schwann cell macrophage” paracrine axis. Notably, the PR inhibitor RU486 only partially attenuated M2 polarization (Figure 2B), hinting at the involvement of parallel pathways beyond classical progesterone signaling. ETX is not only a ligand for TSPO but also a positive allosteric modulator of the GABA-A receptor (Poisbeau et al., 2018). To delineate the contributions of these potential mechanisms, we conducted further investigations. The TSPO-selective ligand Ro5-4864 mimicked the effects of ETX, confirming that TSPO activation is sufficient to initiate this cascade. To assess whether GABA-A receptor signaling contributes to ETX’s effects, we employed the antagonist Gabazine. Results showed that Gabazine only partially attenuated ETX-induced M2 polarization, an effect less pronounced than that observed with RU486. This suggests that ETX operates within a broader, multi-target synergistic network, rather than through a single linear pathway. While GABAergic signaling may have a limited direct contribution to this specific phenotypic switch in our simplified coculture system, its potential indirect regulatory roles in the complex *in vivo* environment—such as modulating neuronal excitability or Schwann cell function—cannot be entirely excluded. Furthermore, the partial inhibition by both RU486 and Gabazine collectively indicates that the pro-repair effects of ETX are likely an integrated outcome of its action on multiple cell types and multiple targets. Secondly, although the Transwell co-culture system employed in this study effectively simulates paracrine communication between cells, its design inherently cannot precisely distinguish the initial cellular target of ETX. In this system, the drug is present in both the upper and lower chambers, and thus can act on Schwann cells while also directly contacting macrophages. Therefore, the enhanced M2 polarization phenotype we observed cannot be solely attributed to a single pathway in a single cell type, but is more likely the integrated outcome of ETX acting on this interconnected cellular network. This limitation suggests that the therapeutic effects of ETX in the *in vivo* environment may involve an even more complex network of intercellular interactions, potentially including ancillary contributions from GABA_A receptor modulation and progesterone metabolites that synergistically contribute to the overall therapeutic benefit.

RNA-seq and functional validation revealed that Pg drives M2 polarization by activating the PPARγ signaling pathway in macrophages, while previous studies have reported that progesterone significantly upregulates PPARγ expression in the liver (including both the maternal and fetal liver) by activating its receptor PR-B (Jeong et al., 2024). PPARγ activation is closely associated with fatty acid oxidation, and PPARγ transcriptionally activates fatty acid oxidation (FAO)-related genes to drive mitochondrial fatty acid β-oxidation, constituting a pivotal pathway for regulating muscle regeneration and energy metabolism (Luo et al., 2023). In our study, we quantitatively assessed PPARγ expression and fatty acid oxidation (FAO) activity. Our results show that progesterone (Pg) significantly upregulates the expression of PPARγ, increases the expression of its coactivator PGC1α, and increases both the activity of CPT1A (a rate-limiting FAO enzyme) and overall FAO capacity. Critically, PPARγ-mediated FAO activation directly regulates the polarization of macrophages toward the M2 phenotype (Liu et al., 2021). PPARγ agonists induce the expression of Arg1 and various transport proteins (FABP and CD36) in macrophages and promote their polarization toward an alternative M2-like phenotype (Peng et al., 2025). Given that the progesterone metabolite allopregnanolone has also been reported to possess PPARγ-activating potential (Wang et al., 2020), we further investigated the signal source driving PPARγ activation in macrophages in our system. Our results show that while ETX treatment promoted allopregnanolone production in the co-culture system, blocking the conversion of progesterone to allopregnanolone with dutasteride only partially reduced PPARγ expression. In contrast, direct blockade of the progesterone receptor with RU486 abrogated PPARγ upregulation to a greater extent. This comparison suggests that although allopregnanolone may exert a synergistic effect, progesterone itself, acting through its classical nuclear receptor (PR) to regulate PPARγ expression, constitutes the primary mechanism in this signaling axis, and this process does not entirely depend on its conversion to allopregnanolone. Our data further suggest that the pro-reparative effect of ETX may not be entirely dependent on the classical progesterone receptor (PR) pathway. We found that treatment with either the progesterone receptor antagonist RU486 or the 5α-reductase inhibitor Dutasteride (which blocks the conversion of progesterone to Allopregnanolone) only partially inhibited the ETX-induced M2 polarization effect. Combined with the observation that treatment with the GABA_A receptor antagonist Gabazine also produced a mild inhibitory effect (Figures 2C,D), these results indicate that the biological effects of ETX are likely mediated by multiple signaling pathways. On one hand, the activation of PPARγ by progesterone itself through its classical nuclear receptor PR is the dominant mechanism; on the other hand, allopregnanolone, a metabolite of progesterone, may play a synergistic or complementary role in the maintenance of M2 polarization by activating GABA_A receptors or directly acting on PPARγ. This mechanistic redundancy precisely reflects the robustness of biological systems in responding to injury. The process of fatty acid oxidation provides ATP for macrophages, supporting the energy-intensive functions of M2 polarization, while its metabolic byproducts acetyl-CoA and NADH increase the expression of M2-associated genes (Huang et al., 2016).

This study further confirmed that the activation of PPARγ significantly upregulated the expression of mitophagy-related proteins, thereby triggering mitophagy. Using the mito-QC reporter system, we observed a significant increase in mCherry^+^GFP^–^ puncta, which represent autolysosomes, and this effect was blocked by the PPARγ antagonist GW9662. The mitophagy induced in our system likely plays a crucial biological role. In parallel with the observed upregulation of mitophagy markers (BNIP3L, Parkin) and autophagic flux (increased LC3-II, decreased p62; Figures 4B–G), we noted a significant decrease in the inflammasome component NLRP3 (Figure 4D). This aligns with the established concept that accelerated FAO can increase mitochondrial stress, and that clearance of dysfunctional mitochondria via mitophagy prevents mtDNA/NLRP3 inflammasome activation (Ko et al., 2021; Chen et al., 2025). Thus, our data suggest that PPARγ-driven mitophagy (Figures 4I–K) contributes to maintaining the M2 phenotype by concurrently suppressing a key inflammatory pathway (NLRP3 reduction) and potentially preserving the metabolic fitness of mitochondria required for sustained FAO. However, regarding the relationship between PPARγ and mitophagy, no consensus has been reached. Previous studies have demonstrated that in diffuse large B-cell lymphoma (DLBCL), PPARγ interacts with PINK1, activating the PINK1–Parkin pathway, enhancing mitophagy and ultimately leading to tumor cell death (Zhou et al., 2025). Another study also revealed that PPARγ activation upregulates PINK1 expression and promotes Parkin phosphorylation at Ser65, thereby enhancing PINK1/Parkin-mediated mitophagy. This process clears damaged mitochondria, improves mitochondrial function and increases ATP production, ultimately enhancing cerebral glucose metabolism, reducing Aβ deposition, and ameliorating cognitive function in Alzheimer’s disease model mice (Li Z. et al., 2022). Conversely, studies of pulmonary arterial hypertension (PH) have shown that SMYD2 suppresses the transcriptional activity of PPARγ and then activates FUNDC1-mediated mitophagy. This process leads to mitochondrial dysfunction, ultimately promoting the excessive proliferation of pulmonary arterial smooth muscle cells (PASMCs), pulmonary vascular remodeling, and the development of PH (Li et al., 2024). Notably, the phenomenon of PPARγ promoting macrophage mitophagy observed in this study may be associated with its role in regulating macrophage polarization toward the M2 phenotype.

Animal experiments validated the therapeutic effects of ETX and its underlying mechanisms. In a sciatic nerve crush injury model, ETX administration significantly promoted functional recovery in rats, as evidenced by sustained improvements in hindlimb grasping force and mechanical pain threshold. More importantly, in-depth mechanistic validation demonstrated that ETX functions via the proposed “Schwann cell–macrophage” paracrine axis. Immunofluorescence analysis on day 7 post-injury revealed that ETX treatment effectively increased the infiltration of pro-regenerative M2-type macrophages (CD68^+^CD206^+^) within the injured nerve. This effect could be attenuated by co-administration of the progesterone receptor inhibitor RU486 or the PPARγ inhibitor GW9662, providing strong evidence for the central role of the Pg-PPARγ signaling axis in driving M2 polarization *in vivo*. Further analysis at the protein level showed that ETX treatment significantly upregulated PPARγ expression at the injury site, while the autophagy substrate p62 was decreased, consistent with enhanced autophagic flux. Moreover, the increased co-expression of PPARγ in macrophages was partially reversed by RU486, thereby linking Schwann cell-derived Pg to the activation of the PPARγ pathway within macrophages *in vivo*. Concomitant with these immunological and molecular changes, structural regeneration was substantially promoted. Retrograde tracing on day 15 post-injury demonstrated a significantly greater number of labeled regenerated neurons in the spinal cord of ETX-treated rats compared to the control group. Simultaneously, the extent of NF200^+^ axonal regeneration was more extensive. Collectively, these results indicate that ETX, by activating the “Schwann cell-Pg-macrophage PPARγ” signaling axis to drive M2 macrophage polarization, thereby promotes both functional and structural nerve repair.

This study has several limitations. First, although THP-1 monocyte-derived macrophages are a widely accepted cell model, they may not fully recapitulate the metabolic and functional characteristics of primary macrophages *in vivo*. Future studies employing primary macrophages will help to enhance the generalizability of the proposed signaling pathway.

Second, progesterone may not be the only active factor secreted by Schwann cells in response to ETX. We observed that the dynamic coculture system induced M2 polarization more effectively than static conditioned medium, suggesting that other soluble mediators, induced by bidirectional intercellular communication, may act synergistically with progesterone. Studies have confirmed that Schwann cells can secrete a variety of cytokines and chemokines after nerve injury to directly regulate macrophage recruitment and polarization, including IL-17B (Huang et al., 2024), MFG-E8-containing exosomes (Ren et al., 2023), sFRP1 (Yao et al., 2024), and IL-34 (Si et al., 2024). Furthermore, under hypoxic conditions, recruited macrophages induce angiogenesis by secreting VEGF-A, and these newly formed blood vessels provide guiding scaffolds for Schwann cell migration, forming a “blood vessel-Schwann cell” cooperative regeneration unit (Cattin et al., 2015). Together, these findings reveal a complex paracrine communication network between Schwann cells and macrophages, suggesting that the factors mentioned above may collaborate with progesterone to construct a pro-regenerative “metabolic-immune microenvironment.” Future studies should employ methods such as proteomics to systematically identify these synergistic factors, in order to more comprehensively map the molecular landscape of the Schwann cell-macrophage metabolic dialogue.

Finally, potential species differences warrant attention. The *in vitro* experiments in this study used human cell lines, whereas the *in vivo* validation was conducted in a rat model. Although core pathways are generally conserved, interspecies differences in TSPO ligand affinity, progesterone metabolism, or immune responses may affect translational relevance. These considerations are important for the future clinical application of these findings.

In summary, this study establishes the central role of the progesterone-PPARγ axis in ETX-promoted nerve repair, while also recognizing the complex regulatory network potentially constituted by GABA_A receptors, progesterone metabolites, and other paracrine factors. This coexistence of multiple mechanisms provides a theoretical basis for amplifying therapeutic efficacy through combination therapies in the future, such as ETX combined with PPARγ agonists.

Based on these findings, we propose three key directions for future research: (1) screening other TSPO ligands for similar nerve repair capabilities; (2) evaluating the synergistic potential of PPARγ agonists (such as rosiglitazone) in combination with ETX therapy; and (3) validating the efficacy of ETX in chronic disease models, particularly diabetic peripheral neuropathy.

This study proposes a novel concept of “Schwann cell-macrophage metabolic crosstalk” and elucidates the primary mechanism by which ETX promotes nerve regeneration. Although our data emphasize the dominant role of the TSPO-progesterone-PPARγ axis in driving pro-regenerative macrophage polarization, the potential ancillary contribution of GABA_A receptor-mediated effects on neuronal or glial function in the broader *in vivo* context also warrants further investigation.

## Conclusion

This study identifies a core signaling axis by which the TSPO ligand Etifoxine (ETX) accelerates peripheral nerve repair. The process is initiated when ETX activates TSPO on Schwann cells, stimulating them to synthesize and secrete progesterone. This Schwann cell-derived progesterone then acts as a paracrine signal on macrophages, binding to their progesterone receptors and activating the PPARγ–PGC1α signaling axis. This activation triggers a dual response in the macrophages: a metabolic reprograming toward fatty acid oxidation (FAO) to fuel their energetic demands, and the induction of BNIP3L-mediated mitophagy to clear damaged mitochondria and maintain metabolic homeostasis. The synergy of this metabolic-autophagic reprograming is critical for sustaining a pro-regenerative M2 macrophage polarization.

Importantly, our data also reveal that this progesterone-driven PPARγ axis operates within a broader, multi-target synergistic network. The partial inhibitory effects of the GABA_A receptor antagonist Gabazine and the 5α-reductase inhibitor Dutasteride suggest that GABAergic signaling and progesterone metabolites (such as allopregnanolone) may contribute as ancillary or modulatory inputs within the complex *in vivo* environment. Furthermore, the enhanced efficiency of the dynamic coculture system points to the existence of other, yet-to-be-identified paracrine factors that participate in the Schwann cell-macrophage dialogue. Thus, while the TSPO-progesterone-PPARγ pathway constitutes the central pillar of ETX’s pro-regenerative action, it is likely embedded within a complex regulatory network that collectively orchestrates the regenerative response.

Ultimately, this orchestrated “Schwann cell-macrophage metabolic crosstalk” enhances axonal regeneration, remyelination, and functional recovery after nerve injury. These findings not only reveal a critical pathway for neurosteroid-driven nerve repair but also highlight the inherent complexity of cell-cell metabolic interactions, strongly supporting the therapeutic repurposing of ETX and the potential of targeting the PPARγ pathway for treating peripheral nerve injuries.

## Data Availability

The RNA-seq data generated in this study have been deposited in the National Center for Biotechnology Information (NCBI) BioProject database under accession number PRJNA1403785. The associated raw sequence reads are accessible through the Sequence Read Archive (SRA) under the same BioProject ID.

## Supplementary material

Supplement Figure 1 **(A)** Flow cytometry plots of CD68-APC and CD206-PE. Cells were cultured under four conditions: i. Control (macrophages alone); ii. Co-culture with Ro54864 (HSCs + macrophages + Ro54864); iii. Ro54864 (macrophages + Ro54864); iv. Co-culture medium. **(B)** Quantification of CD68 + CD206 + cells in **(A)**. Data are presented as means ± SEMs; *n* = 3; **P* < 0.05, ***P* < 0.01 (one-way ANOVA, Tukey’s test). **(C)** Effect of progesterone on macrophage polarization. Left panel: Flow cytometry plots of CD68-APC and CD206-PE (Control vs. Progesterone). Right panel: Quantification of CD68 + CD206 + cells. Data are presented as means ± SEMs; *n* = 3; **P* < 0.05 (Student’s *t*-test). (D) Effect of CM-ETX (ETX-conditioned medium) on macrophage polarization. Left panel: Flow cytometry plots of CD68-APC and CD206-PE. Right panel: Quantification of CD68 + CD206 + cells. Data are presented as means ± SEMs; *n* = 3; **P* < 0.05 (Student’s *t*-test). **(E)** Western blot analysis of TSPO knockdown. Cell lysates were collected from HSC transfected with shControl or shTSPO. β-actin was used as a loading control.

## References

[B1] Barresi E. Robello M. Costa B. Da Pozzo E. Baglini E. Salerno S. et al. (2021). An update into the medicinal chemistry of translocator protein (TSPO) ligands. *Eur. J. Med. Chem.* 209:112924. 10.1016/j.ejmech.2020.112924 33081988

[B2] Bonsack F. Alleyne C. H. Sukumari-Ramesh S. (2016). Augmented expression of TSPO after intracerebral hemorrhage: A role in inflammation? *J. Neuroinflammation* 13:151. 10.1186/s12974-016-0619-2 27315802 PMC4912814

[B3] Brosius Lutz A. Lucas T. A. Carson G. A. Caneda C. Zhou L. Barres B. A. et al. (2022). An RNA-sequencing transcriptome of the rodent Schwann cell response to peripheral nerve injury. *J. Neuroinflammation* 19:105. 10.1186/s12974-022-02462-6 35501870 PMC9063194

[B4] Cattin A.-L. Burden J. J. Van Emmenis L. Mackenzie F. E. Hoving J. J. A. Garcia Calavia N. et al. (2015). Macrophage-induced blood vessels guide Schwann cell-mediated regeneration of peripheral nerves. *Cell* 162 1127–1139. 10.1016/j.cell.2015.07.021 26279190 PMC4553238

[B5] Chen L. Hu P. Hong X. Li B. Ping Y. Chen S. et al. (2025). Dimethyl fumarate modulates M1/M2 macrophage polarization to ameliorate periodontal destruction by increasing TUFM-mediated mitophagy. *Int. J. Oral Sci.* 17:32. 10.1038/s41368-025-00360-0 40246816 PMC12006468

[B6] Gaudet A. D. Popovich P. G. Ramer M. S. (2011). Wallerian degeneration: Gaining perspective on inflammatory events after peripheral nerve injury. *J. Neuroinflammation* 8:110. 10.1186/1742-2094-8-110 21878126 PMC3180276

[B7] Girard C. Liu S. Cadepond F. Adams D. Lacroix C. Verleye M. et al. (2008). Etifoxine improves peripheral nerve regeneration and functional recovery. *Proc. Natl. Acad. Sci. U.S.A.* 105 20505–20510. 10.1073/pnas.0811201106 19075249 PMC2629330

[B8] Huang S. C.-C. Smith A. M. Everts B. Colonna M. Pearce E. L. Schilling J. D. et al. (2016). Metabolic reprogramming mediated by the mTORC2-IRF4 signaling axis is essential for macrophage alternative activation. *Immunity* 45 817–830. 10.1016/j.immuni.2016.09.016 27760338 PMC5535820

[B9] Huang Y. Wu L. Zhao Y. Guo J. Li R. Ma S. et al. (2024). Schwann cell promotes macrophage recruitment through IL-17B/IL-17RB pathway in injured peripheral nerves. *Cell Rep.* 43:113753. 10.1016/j.celrep.2024.113753 38341853

[B10] Jeong K. J. Mukae M. Lee S. R. Kim S.-Y. Kim S. H. Cho Y.-E. et al. (2024). Progesterone increases hepatic lipid content and plasma lipid levels through PR- B-mediated lipogenesis. *Biomed. Pharmacother.* 172:116281. 10.1016/j.biopha.2024.116281 38364736

[B11] Ko M. S. Yun J. Y. Baek I.-J. Jang J. E. Hwang J. J. Lee S. E. et al. (2021). Mitophagy deficiency increases NLRP3 to induce brown fat dysfunction in mice. *Autophagy* 17 1205–1221. 10.1080/15548627.2020.1753002 32400277 PMC8143238

[B12] Lacapère J.-J. Papadopoulos V. (2003). Peripheral-type benzodiazepine receptor: Structure and function of a cholesterol-binding protein in steroid and bile acid biosynthesis. *Steroids* 68 569–585. 10.1016/S0039-128X(03)00101-6 12957662

[B13] Li J. Yao Y. Wang Y. Xu J. Zhao D. Liu M. et al. (2022). Modulation of the crosstalk between Schwann cells and macrophages for nerve regeneration: A therapeutic strategy based on a multifunctional tetrahedral framework nucleic acids system. *Adv. Mater.* 34:e2202513. 10.1002/adma.202202513 35483031

[B14] Li Y. Lv S. Yuan H. Ye G. Mu W. Fu Y. et al. (2021). Peripheral nerve regeneration with 3d printed bionic scaffolds loading neural crest stem cell derived Schwann cell progenitors. *Adv. Funct. Mater.* 31:2010215. 10.1002/adfm.202010215

[B15] Li Y. Wei X. Xiao R. Chen Y. Xiong T. Fang Z.-M. et al. (2024). SMYD2-Methylated PPARγ facilitates hypoxia-induced pulmonary hypertension by activating mitophagy. *Circ. Res.* 135 93–109. 10.1161/CIRCRESAHA.124.323698 38770649

[B16] Li Z. Meng X. Ma G. Liu W. Li W. Cai Q. et al. (2022). Increasing brain glucose metabolism by ligustrazine piperazine ameliorates cognitive deficits through PPARγ-dependent enhancement of mitophagy in APP/PS1 mice. *Alzheimers Res. Ther.* 14:150. 10.1186/s13195-022-01092-7 36217155 PMC9552451

[B17] Liu P. Peng J. Han G.-H. Ding X. Wei S. Gao G. et al. (2019). Role of macrophages in peripheral nerve injury and repair. *Neural Regen. Res.* 14 1335–1342. 10.4103/1673-5374.253510 30964051 PMC6524518

[B18] Liu S. Zhang H. Li Y. Zhang Y. Bian Y. Zeng Y. et al. (2021). S100A4 enhances protumor macrophage polarization by control of PPAR-γ-dependent induction of fatty acid oxidation. *J. Immunother. Cancer* 9 e002548. 10.1136/jitc-2021-002548 34145030 PMC8215236

[B19] Luo L. Chua Y.-J. B. Liu T. Liang K. Chua M.-W. J. Ma W. et al. (2023). Muscle injuries induce a prostacyclin-PPARγ/PGC1a-FAO spike that boosts regeneration. *Adv. Sci. Weinh. Baden Wurtt. Ger.* 10:e2301519. 10.1002/advs.202301519 37140179 PMC10375192

[B20] Mahar M. Cavalli V. (2018). Intrinsic mechanisms of neuronal axon regeneration. *Nat. Rev. Neurosci.* 19 323–337. 10.1038/s41583-018-0001-8 29666508 PMC5987780

[B21] Mildner A. Marinkovic G. Jung S. (2016). Murine monocytes: Origins, subsets, fates, and functions. *Microbiol. Spectr.* 4:MCHD-0033-2016. 10.1128/microbiolspec.MCHD-0033-2016 27780020

[B22] Monga S. Nagler R. Amara R. Weizman A. Gavish M. (2019). Inhibitory effects of the two novel TSPO Ligands 2-Cl-MGV-1 and MGV-1 on LPS-induced microglial activation. *Cells* 8:486. 10.3390/cells8050486 31121852 PMC6562711

[B23] Nadeau S. Filali M. Zhang J. Kerr B. J. Rivest S. Soulet D. et al. (2011). Functional recovery after peripheral nerve injury is dependent on the pro-inflammatory cytokines IL-1β and TNF: Implications for neuropathic pain. *J. Neurosci.* 31 12533–12542. 10.1523/JNEUROSCI.2840-11.2011 21880915 PMC6703268

[B24] Papadopoulos V. Baraldi M. Guilarte T. R. Knudsen T. B. Lacapère J.-J. Lindemann P. et al. (2006). Translocator protein (18kDa): New nomenclature for the peripheral-type benzodiazepine receptor based on its structure and molecular function. *Trends Pharmacol. Sci.* 27 402–409. 10.1016/j.tips.2006.06.005 16822554

[B25] Peng M. Li N. Wang H. Li Y. Liu H. Luo Y. et al. (2025). Macrophages: Subtypes, distribution, polarization, immunomodulatory functions, and therapeutics. *MedComm* 6:e70304. 10.1002/mco2.70304 40717900 PMC12290311

[B26] Poisbeau P. Gazzo G. Calvel L. (2018). Anxiolytics targeting GABAA receptors: insights on etifoxine. *World J Biol Psychiatry.* 19, S36–S45. 10.1080/15622975.2018.1468030 30204559

[B27] Ren J. Zhu B. Gu G. Zhang W. Li J. Wang H. et al. (2023). Schwann cell-derived exosomes containing MFG-E8 modify macrophage/microglial polarization for attenuating inflammation via the SOCS3/STAT3 pathway after spinal cord injury. *Cell Death Dis.* 14:70. 10.1038/s41419-023-05607-4 36717543 PMC9887051

[B28] Rupprecht R. Wetzel C. H. Dorostkar M. Herms J. Albert N. L. Schwarzbach J. et al. (2022). Translocator protein (18kDa) TSPO: A new diagnostic or therapeutic target for stress-related disorders? *Mol. Psychiatry* 27 2918–2926. 10.1038/s41380-022-01561-3 35444254

[B29] Si W. Chen Z. Bei J. Chang S. Zheng Y. Gao L. et al. (2024). Stigmasterol alleviates neuropathic pain by reducing Schwann cell-macrophage cascade in DRG by modulating IL-34/CSF1R. *CNS Neurosci. Ther.* 30:e14657. 10.1111/cns.14657 38572785 PMC10993342

[B30] Soto P. A. Vence M. Piñero G. M. Coral D. F. Usach V. Muraca D. et al. (2021). Sciatic nerve regeneration after traumatic injury using magnetic targeted adipose-derived mesenchymal stem cells. *Acta Biomater.* 130 234–247. 10.1016/j.actbio.2021.05.050 34082099

[B31] Viola A. Munari F. Sánchez-Rodríguez R. Scolaro T. Castegna A. (2019). The metabolic signature of macrophage responses. *Front. Immunol.* 10:1462. 10.3389/fimmu.2019.01462 31333642 PMC6618143

[B32] Wang T. Yao J. Chen S. Mao Z. Brinton R. D. (2020). Allopregnanolone reverses bioenergetic deficits in female triple transgenic Alzheimer’s mouse model. *Neurotherapeutics* 17 178–188. 10.1007/s13311-019-00793-6 31664643 PMC7053503

[B33] Yao X. Kong L. Qiao Y. Brand D. Li J. Yan Z. et al. (2024). Schwann cell-secreted frizzled-related protein 1 dictates neuroinflammation and peripheral nerve degeneration after neurotrauma. *Cell Rep. Med.* 5:101791. 10.1016/j.xcrm.2024.101791 39426375 PMC11604536

[B34] Yin J. Huang J. Wu J. Gao L. Xu H. Zhu J. et al. (2025). Emapunil relieves osteoarthritis by regulating the CD14/TLR4/LY96 pathway in synovial macrophages through translocator protein 18 kDa. *Osteoarthritis Cartilage* 33 1107–1120. 10.1016/j.joca.2025.06.008 40541821

[B35] Zhou X. Guo Q. Qiao Q. Fang X. Jiang Y. Wang X. et al. (2025). Magnolin promotes PINK1-parkin-mediated mitophagy in diffuse large B-cell lymphoma cells via PPAR-γ pathway. *Phytomedicine* 145:157059. 10.1016/j.phymed.2025.157059 40680330

[B36] Zigmond R. E. Echevarria F. D. (2019). Macrophage biology in the peripheral nervous system after injury. *Prog. Neurobiol.* 173 102–121. 10.1016/j.pneurobio.2018.12.001 30579784 PMC6340791

