# Supplementary material for: Etifoxine drives macrophage M2 polarization via Schwann cell-derived progesterone activation of PPARγ to accelerate peripheral nerve repair
Source: Front Cell Neurosci. 2026 Mar 23;20:1789450. doi: 10.3389/fncel.2026.1789450 (PMC13060035; doi:10.3389/fncel.2026.1789450)
Supplement: Supplementary file 1 [file Supplementary_file_1.docx]

**Document 1: Detailed Behavioral Data on Functional Recovery After Rat Sciatic Nerve Crush Injury**

**1. Objective**
To perform detailed pairwise comparisons of functional recovery outcomes between treatment groups (Control, ETX, RU486+ETX, GW9662+ETX) at each observed time point (Day 0, Day 3, Day 7, Day 15) following rat sciatic nerve crush injury. Two functional parameters were assessed: (1) hindlimb grip strength (reflecting motor function recovery) and (2) mechanical pain threshold (reflecting sensory function recovery). These data correspond to the behavioral results summarized in Figure 5A-B.

**2. Statistical Methods**
Data were analyzed using two-way repeated measures ANOVA (factors: time and treatment), followed by Tukey's post-hoc test for multiple comparisons. This analysis aimed to examine the simple main effects of treatment within each time point. A family-wise error rate α = 0.05 was set as the threshold for statistical significance. Adjusted p-values (q-values) are reported to account for the 6 comparisons performed at each time point. Results are presented as mean differences with 95% confidence intervals (CI). All statistical analyses were performed using GraphPad Prism 9.0.

**3. Results**

**3.1 Hindlimb Grip Strength (Motor Function Recovery)**
Tukey's post-hoc analysis of hindlimb grip strength revealed time-dependent treatment effects (Supplementary Table 1).

**Day 7:**

The ETX group showed significantly increased grip strength compared to the Control group (Mean Difference = -6.103, 95% CI [-11.23, -0.980], p = 0.0324).The RU486+ETX group exhibited significantly lower grip strength compared to both the Control group (Mean Difference = 2.8, 95% CI [0.671, 4.929], p = 0.02) and the ETX group (Mean Difference = 8.903, 95% CI [3.678, 14.13], p = 0.0135).The GW9662+ETX group also showed significantly lower grip strength compared to the ETX group (Mean Difference = 7.383, 95% CI [2.286, 12.48], p = 0.0195).

**Day 15:** The ETX group maintained significantly higher grip strength compared to the Control group (Mean Difference = -9.7, 95% CI [-18.52, -0.876], p = 0.038), the RU486+ETX group (Mean Difference = 12.86, 95% CI [3.730, 21.99], p = 0.0199), and the GW9662+ETX group (Mean Difference = 9.787, 95% CI [0.194, 19.38], p = 0.0477).

**Supplementary Table 1. Tukey's multiple comparisons test for hindlimb grip strength at different time points**

| Time Point | Comparison | Mean Difference | 95% CI | Adjusted P Value | Significance |
| --- | --- | --- | --- | --- | --- |
| Day 0 | Control vs. ETX | 0.05667 | -2.182 to 2.295 | 0.9995 | ns |
| Day 0 | Control vs. RU486+ETX | 2.083 | 0.09734 to 4.069 | 0.0437 | * |
| Day 0 | Control vs. GW9662+ETX | 1.243 | -0.9488 to 3.436 | 0.1714 | ns |
| Day 0 | ETX vs. RU486+ETX | 2.027 | -0.1360 to 4.189 | 0.0595 | ns |
| Day 0 | ETX vs. GW9662+ETX | 1.187 | -1.213 to 3.587 | 0.2174 | ns |
| Day 0 | RU486+ETX vs. GW9662+ETX | -0.84 | -2.004 to 0.3239 | 0.1202 | ns |
| Day 3 | Control vs. ETX | -0.06333 | -2.370 to 2.243 | 0.9991 | ns |
| Day 3 | Control vs. RU486+ETX | 2.273 | -0.06850 to 4.615 | 0.0549 | ns |
| Day 3 | Control vs. GW9662+ETX | 1.033 | -1.291 to 3.358 | 0.3781 | ns |
| Day 3 | ETX vs. RU486+ETX | 2.337 | 0.3890 to 4.284 | 0.0288 | * |
| Day 3 | ETX vs. GW9662+ETX | 1.097 | -0.7941 to 2.987 | 0.2105 | ns |
| Day 3 | RU486+ETX vs. GW9662+ETX | -1.24 | -3.338 to 0.8578 | 0.2174 | ns |
| Day 7 | Control vs. ETX | -6.103 | -11.23 to -0.9804 | 0.0324 | * |
| Day 7 | Control vs. RU486+ETX | 2.8 | 0.6708 to 4.929 | 0.02 | * |
| Day 7 | Control vs. GW9662+ETX | 1.28 | -0.9561 to 3.516 | 0.2339 | ns |
| Day 7 | ETX vs. RU486+ETX | 8.903 | 3.678 to 14.13 | 0.0135 | * |
| Day 7 | ETX vs. GW9662+ETX | 7.383 | 2.286 to 12.48 | 0.0195 | * |
| Day 7 | RU486+ETX vs. GW9662+ETX | -1.52 | -3.680 to 0.6399 | 0.1401 | ns |
| Day 15 | Control vs. ETX | -9.7 | -18.52 to -0.8758 | 0.038 | * |
| Day 15 | Control vs. RU486+ETX | 3.16 | -2.429 to 8.749 | 0.2332 | ns |
| Day 15 | Control vs. GW9662+ETX | 0.08667 | -5.446 to 5.619 | 0.9999 | ns |
| Day 15 | ETX vs. RU486+ETX | 12.86 | 3.730 to 21.99 | 0.0199 | * |
| Day 15 | ETX vs. GW9662+ETX | 9.787 | 0.1940 to 19.38 | 0.0477 | * |
| Day 15 | RU486+ETX vs. GW9662+ETX | -3.073 | -7.529 to 1.383 | 0.145 | ns |

**3.2 Mechanical Pain Threshold (Sensory Function Recovery)**
Tukey's post-hoc analysis of the mechanical pain threshold also revealed clear time-dependent treatment effects (Supplementary Table 2).

**Day 7:**

Compared to the Control group, the ETX group exhibited a significantly increased mechanical pain threshold (Mean Difference = 1.67, 95% CI [0.619, 2.721], p = 0.0103), indicating improved sensory recovery.Conversely, the GW9662+ETX group showed a significantly decreased mechanical pain threshold compared to the Control group (Mean Difference = -1.93, 95% CI [-2.838, -1.022], p = 0.0037).Furthermore, compared to the ETX group, both the RU486+ETX group (Mean Difference = -2.227, 95% CI [-3.284, -1.169], p = 0.0036) and the GW9662+ETX group (Mean Difference = -3.6, 95% CI [-4.615, -2.585], p = 0.0007) had significantly lower mechanical pain thresholds. The RU486+ETX group was also significantly lower than the GW9662+ETX group (Mean Difference = -1.373, 95% CI [-2.295, -0.452], p = 0.0133).

**Day 15:**

The ETX group maintained a significantly higher mechanical pain threshold compared to the Control group (Mean Difference = 1.32, 95% CI [0.366, 2.274], p = 0.0168).Compared to the ETX group, both the RU486+ETX group (Mean Difference = -1.633, 95% CI [-2.580, -0.686], p = 0.0077) and the GW9662+ETX group (Mean Difference = -1.417, 95% CI [-2.331, -0.502], p = 0.0125) showed significantly lower mechanical pain thresholds.

**Supplementary Table 2. Tukey's multiple comparisons test for mechanical pain threshold at different time points**

| Time Point | Comparison | Mean Difference | 95% CI | Adjusted P Value | Significance |
| --- | --- | --- | --- | --- | --- |
| Day 0 | Control vs. ETX | -0.2967 | -2.846 to 2.253 | 0.9179 | ns |
| Day 0 | Control vs. RU486+ETX | -0.05 | -1.265 to 1.165 | 0.9978 | ns |
| Day 0 | Control vs. GW9662+ETX | -0.1 | -1.123 to 0.9233 | 0.976 | ns |
| Day 0 | ETX vs. RU486+ETX | 0.2467 | -2.160 to 2.654 | 0.9551 | ns |
| Day 0 | ETX vs. GW9662+ETX | 0.1967 | -2.343 to 2.736 | 0.9724 | ns |
| Day 0 | RU486+ETX vs. GW9662+ETX | -0.05 | -1.268 to 1.168 | 0.9979 | ns |
| Day 3 | Control vs. ETX | -0.1633 | -1.240 to 0.9131 | 0.9211 | ns |
| Day 3 | Control vs. RU486+ETX | 0.4367 | -0.5871 to 1.460 | 0.4129 | ns |
| Day 3 | Control vs. GW9662+ETX | -0.06667 | -1.263 to 1.130 | 0.9949 | ns |
| Day 3 | ETX vs. RU486+ETX | 0.6 | -0.4530 to 1.653 | 0.2349 | ns |
| Day 3 | ETX vs. GW9662+ETX | 0.09667 | -1.113 to 1.306 | 0.9859 | ns |
| Day 3 | RU486+ETX vs. GW9662+ETX | -0.5033 | -1.688 to 0.6814 | 0.4005 | ns |
| Day 7 | Control vs. ETX | 1.67 | 0.6188 to 2.721 | 0.0103 | * |
| Day 7 | Control vs. RU486+ETX | -0.5567 | -1.539 to 0.4257 | 0.2393 | ns |
| Day 7 | Control vs. GW9662+ETX | -1.93 | -2.838 to -1.022 | 0.0037 | ** |
| Day 7 | ETX vs. RU486+ETX | -2.227 | -3.284 to -1.169 | 0.0036 | ** |
| Day 7 | ETX vs. GW9662+ETX | -3.6 | -4.615 to -2.585 | 0.0007 | *** |
| Day 7 | RU486+ETX vs. GW9662+ETX | -1.373 | -2.295 to -0.4520 | 0.0133 | * |
| Day 15 | Control vs. ETX | 1.32 | 0.3662 to 2.274 | 0.0168 | * |
| Day 15 | Control vs. RU486+ETX | -0.3133 | -1.198 to 0.5714 | 0.5398 | ns |
| Day 15 | Control vs. GW9662+ETX | -0.09667 | -0.9237 to 0.7304 | 0.9573 | ns |
| Day 15 | ETX vs. RU486+ETX | -1.633 | -2.580 to -0.6864 | 0.0077 | ** |
| Day 15 | ETX vs. GW9662+ETX | -1.417 | -2.331 to -0.5022 | 0.0125 | * |
| Day 15 | RU486+ETX vs. GW9662+ETX | 0.2167 | -0.5952 to 1.028 | 0.7034 | ns |

**4. Summary of Behavioral Findings**
Post-hoc analyses confirmed that ETX treatment significantly promoted motor and sensory functional recovery after sciatic nerve crush injury, as evidenced by sustained improvements in hindlimb grip strength and mechanical pain threshold at later time points (Days 7 and 15) compared to the Control group. These beneficial effects were significantly attenuated by co-treatment with RU486 and GW9662.
