# Supplementary figures and images for: Etifoxine drives macrophage M2 polarization via Schwann cell-derived progesterone activation of PPARγ to accelerate peripheral nerve repair
Source: Front Cell Neurosci. 2026 Mar 23;20:1789450. doi: 10.3389/fncel.2026.1789450 (PMC13060035; doi:10.3389/fncel.2026.1789450)

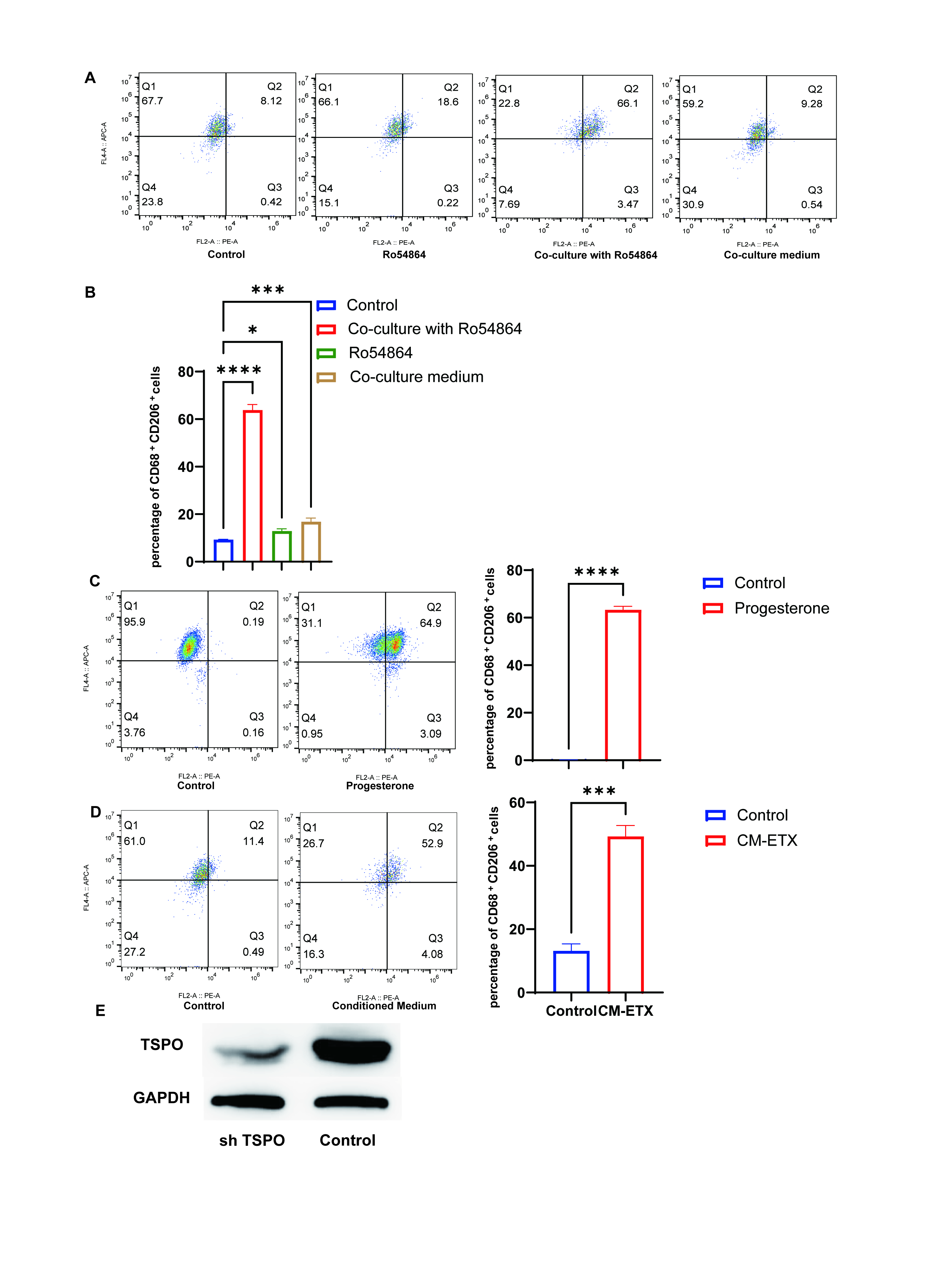

Supplement: Supplement Figure 1 — (A) Flow cytometry plots of CD68-APC and CD206-PE. Cells were cultured under four conditions: i. Control (macrophages alone); ii. Co-culture with Ro54864 (HSCs + macrophages + Ro54864); iii. Ro54864 (macrophages + Ro54864); iv. Co-culture medium. (B) Quantification of CD68 + CD206 + cells in (A). Data are presented as means ± SEMs; n = 3; *P < 0.05, **P < 0.01 (one-way ANOVA, Tukey’s test). (C) Effect of progesterone on macrophage polarization. Left panel: Flow cytometry plots of CD68-APC and CD206-PE (Control vs. Progesterone). Right panel: Quantification of CD68 + CD206 + cells. Data are presented as means ± SEMs; n = 3; *P < 0.05 (Student’s t-test). (D) Effect of CM-ETX (ETX-conditioned medium) on macrophage polarization. Left panel: Flow cytometry plots of CD68-APC and CD206-PE. Right panel: Quantification of CD68 + CD206 + cells. Data are presented as means ± SEMs; n = 3; *P < 0.05 (Student’s t-test). (E) Western blot analysis of TSPO knockdown. Cell lysates were collected from HSC transfected with shControl or shTSPO. β-actin was used as a loading control. [file Image_1.tif]
